# Genome-Wide Identification and Expression Analysis of *ADK* Gene Family Members in Cotton under Abiotic Stress

**DOI:** 10.3390/ijms25147821

**Published:** 2024-07-17

**Authors:** Peijun Huang, Ziwei Lin, Yuzhi Zhang, Yu Gao, Songjuan Tan, Shuai Wang, Xiaoyu Cao, Hongyan Shi, Chao Sun, Jiangping Bai, Xiongfeng Ma

**Affiliations:** 1 College of Agronomy, Gansu Agricultural University, Lanzhou 730070, China; huangpeij12131@163.com (P.H.); sunc@gsau.edu.cn (C.S.); 2 State Key Laboratory of Cotton Bio-Breeding and Integrated Utilization, Institute of Cotton Research, Chinese Academy of Agricultural Sciences, Anyang 455000, China; linziwei1105@163.com (Z.L.); 18839789327@163.com (Y.Z.); gaoyu960618@163.com (Y.G.); tansongjuan@caas.cn (S.T.); wangshuai_19871201@163.com (S.W.); 15393768203@163.com (H.S.); 3 Western Agricultural Research Center, Chinese Academy of Agricultural Sciences, Changji 831100, China; 4 College of Mechanical and Electrical Engineering, Shihezi University, Shihezi 518000, China

**Keywords:** genome-wide, cotton, *ADK* gene family, stress

## Abstract

Adenosine kinase (ADK) is a key enzyme widely distributed in plants, playing an important role in maintaining cellular energy homeostasis and regulating plant growth, development, and responses to environmental stresses. However, research on *ADK* genes in cotton (*Gossypium hirsutum*), an economically significant crop, has been limited. This study identified 92 *ADK* genes from four cotton species (*G. arboreum*, *G. raimondii*, *G. hirsutum*, and *G. barbadense*) using HMMER and Local BLASTP methods and classified them into six groups. Chromosomal localization revealed a random distribution of *ADK* genes in *G. hirsutum*, with 13 genes located on the At subgenome and 14 genes on the Dt subgenome. Gene structure analysis showed consistency in exon–intron organization within subgroups, while conserved motif analysis identified subgroup-specific motifs, indicating functional diversity. Synteny and collinearity mapping analysis revealed that the primary expansion mechanisms of the *ADK* gene family in cotton are polyploidy and segmental duplication. Cis-regulatory elements in *GhADK* promoters were classified into light response, hormone response, developmental regulation, and stress response. We also analyzed the expression patterns of *GhADK* genes under a low temperature (4 °C) and drought conditions. Most *GhADK* genes responded to cold stress with different expression patterns, indicating their roles in rapid response and long-term cold adaptation. Under drought stress, expression patterns varied, with some genes showing sustained high expression levels. The qRT-PCR validation of transcriptomic data confirmed the stress-induced expression patterns of selected *GhADK* genes. Functional analysis through the VIGS silencing of *GhADK25* demonstrated its importance in cold and drought stress responses, with silencing resulting in poor growth under stress, highlighting its significance in stress tolerance. This study provides a basis for further understanding the evolutionary relationships and functions of the cotton *ADK* gene family.

## 1. Introduction

Adenosine monophosphate (AMP), a precursor molecule in the nucleotide metabolism pool, is one of the main mononucleotides that constitute cellular RNA. The formation of AMP is usually accompanied by the release of energy within the organism [1]. The proportions of AMP, adenosine diphosphate (ADP), and adenosine triphosphate (ATP)—the energy molecule and precursor in the carbohydrate metabolism pool—determine the energy charge ratio and twice carbohydrate metabolism, which directly affects plant growth, development, and adaptation to adverse environmental conditions [2,3]. Adenylate kinase (ADK, EC 2.7.4.3) is a universally present and abundant monophosphate transferase found in almost all living organisms [4]. It catalyzes the reversible phosphorylation reaction between ATP and AMP (ATP + AMP ↔ 2ADP) and is considered a key enzyme in maintaining the balance of energy metabolism and various adenylate pools [5,6]. ADK usually consists of an AMP domain, an ATP domain, and a relatively conserved core domain (CORE) [7,8].

Studies have shown that ADK is highly conserved in both animals and plants, and its activity has been confirmed in plants such as Arabidopsis thaliana, *Oryza sativa* (rice), *Zea mays* (maize), *Pisum sativum* (pea), and *Solanum tuberosum* (potato). The subcellular localization of ADK is distributed in different organelles within cells, being reported in the cytoplasm, mitochondria, nucleus, and plastids [9,10,11]. For example, inhibiting *StADK* expression in potato plastids significantly increased the adenylate content and starch yield [12]. In Arabidopsis, T-DNA insertion mutants disrupting the *ADK* gene *At2g37250* exhibited an increased amino acid content and enhanced root growth [13]. Another study indicated that the disruption of the *ADK* gene *At5g47840* led to a loss of chloroplast integrity, causing albino seedlings from the early embryonic to seedling development stages [2]. Additionally, *ADK3* interacts with the chloroplast glyceraldehyde-3-phosphate dehydrogenase, forming a stable complex in green Arabidopsis chloroplasts, which may be a potential mechanism for regulating the crucial ATP-NADPH ratio in the Calvin–Benson cycle [14].

*ADK* plays a regulatory role in plant growth and development and participates extensively in plant responses to abiotic stresses. For instance, when maize roots and stems were treated with solutions of different Ca^2+^/Na^+^ ratios, a significant relationship was found between the *ADK* content and salt stress response [15]. In tomato, gene microarray analysis showed that the expression of an *ADK* homolog (SGN-U214214) was suppressed in salt-treated tissues [16]. Furthermore, other microarray data indicated that the expression of the ADK gene (SGN-U232826) was induced by drought stress in drought-tolerant tomatoes [17]. Using pea seeds as a model, a study on the adenylate balance during seed dehydration and rehydration revealed that *ADK* activity plays a critical role in maintaining the adenylate balance during seed desiccation and maturation [18].

Upland cotton (*Gossypium hirsutum*), a species within the Malvaceae family, originated from South America. Archaeological evidence indicates that it was first discovered in ancient cultures of northern Chile around 3500 to 2500 BCE. In the 15th century, Europeans introduced it to Asia and Africa. Today, it stands as one of the world’s most significant crops and a critical economic crop in China [19]. Abiotic and biotic stresses are the significant challenges in crop production worldwide, and climate change will likely lead to more severe abiotic and biotic stress conditions [20]. Despite the recognized importance of *ADK* genes in regulating plant growth and stress resistance, research on the crucial fiber crop cotton (*Gossypium hirsutum*) is still insufficient [21]. This study identified *ADK* genes from the cotton genome using bioinformatics methods and analyzed their phylogenetic relationships, sequence characteristics, gene localization, chromosomal localization, evolutionary relationships, and cis-elements in promoters. Additionally, using existing transcriptome data, we analyzed the transcriptome of cotton under different abiotic stresses at various time points. We further studied the dynamic expression patterns of the *ADK* family in response to abiotic stresses (such as drought and cold) using qRT-PCR. The function of *GhADK25* under drought stress was validated through virus-induced gene-silencing technology. These findings provide valuable information for the further exploration of the functions and regulatory mechanisms of the *ADK* family in cotton.

This study aims to deepen our understanding of the *ADK* gene family in cotton, including their evolutionary relationships, regulatory mechanisms, and responses under abiotic stress conditions. By elucidating these aspects, we aim to provide valuable insights for enhancing the stress tolerance of cotton crops, thus contributing to sustainable agriculture in the face of climate change.

## 2. Results

### 2.1. Identification and Sequence Analysis of Cotton ADK Genes

Combining the HMMER search and Local BLASTP methods, we totally identified 92 ADK genes in four cotton genomes. *G. arboreum*, *G. raimondii*, *G. hirsutum*, and *G. barbadense* were found to have 16, 14, 30, and 32 ADK proteins, respectively (Additional File S1: Table S1). *G. barbadense* and *G. hirsutum* harbored twice as many ADK proteins as *G. arboretum* and *G. raimondii*. We named the 30 *G. hirsutum* ADK proteins GhADK1–GhADK30 according to their locations on the chromosome. The physicochemical properties of all ADK members in *G. hirsutum* were summarized in Table 1, including the names and IDs of genes, the chromosomal and strand locations, the length of the CDS and amino acid sequence, the molecular weight (MW) and the isoelectric point (pI), and the prediction of subcellular localization. In detail, the GhADKs proteins ranged from 214 aa to 746 aa in length, and the average length was 301 aa, with the predicted molecular weights (MW) being from 24.07 to 83.78 kDa and the theoretical isoelectric points (pI) being from 6.15 to 9.68. The subcellular localization prediction results showed that the localization predictions of 11 ADK proteins were displayed in the cytoplasm, 8 ADK proteins were located in the mitochondria, 7 ADK proteins were located in chloroplast, and the rest were located in the extracellular (2) and membrane-bound chloroplast (2).

### 2.2. Phylogenetic Analysis and Classification of the GhADK Gene Family

**Figure 1:**
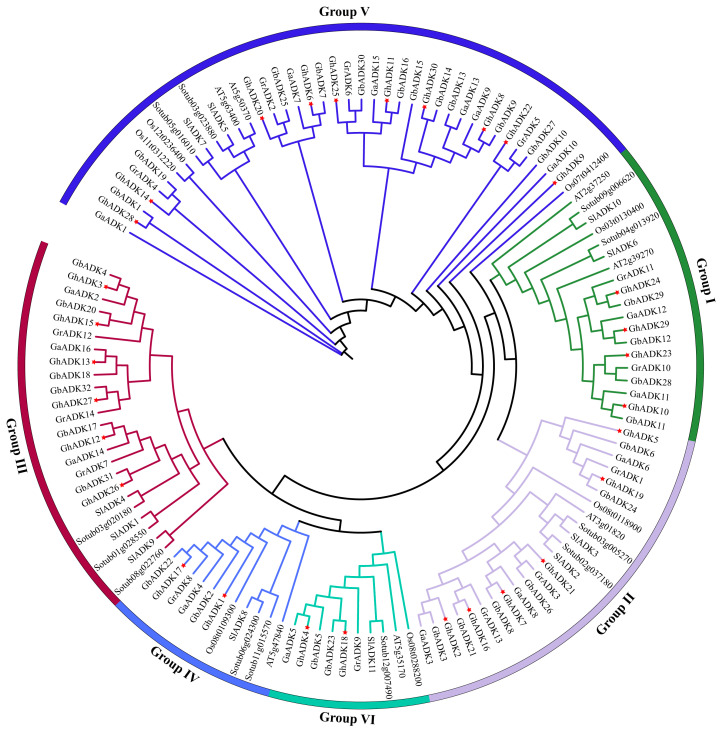
Phylogenetic analysis of cotton species ADK proteins with *A. thaliana*, *O. sativa*, *S. lycopersicum*, and *S. tuberosum*. IQTREE was used to construct the phylogenetic tree using the maximum likelihood method with 1000 bootstrap replications. Each color indicates an individual group (I–VI).

To better understand the evolutionary relationship of GhADKs, we built a phylogenetic tree of the full-length sequences of 92 cotton ADK proteins, 7 Arabidopsis ADK proteins, 7 rice ADK proteins, 11 tomato ADK proteins, and 12 potato ADK proteins (Figure 1).

The phylogenetic analysis revealed that all the ADK proteins were clustered into six groups (Group I–Group VI). The species’ ADK genes were present in almost every clade. Therefore, the subgroups classification was considered more reliable. Group V contained the most ADK members, including ten, six, ten, and four *ADK* genes of *G. hirsutum*, *G. arboreum*, *G. barbadense*, and *G. raimondii*, respectively. As the second largest branch, Group II and Group III contained the same number of family members, with 24 members, including six GhADKs, three GaADKs, six GbADKs, and three GrADKs. Group I has 19 members, representing 14.7% of the total *ADK* genes, including four, two, four, and two ADK genes belonging to *G. hirsutum*, *G. arboreum*, *G. barbadense*, and *G. raimondii*, respectively. Group VI and Group IV occupied ten and eleven ADK members, including two GhADKs, one GaADKs, two GbADKs, and one GrADK. The increase rate of groups II, III, and V in GhADKs is relatively large compared with that in Arabidopsis and other selected species; the expansion of the three groups may have resulted from gene duplications. The phylogenetic analysis results indicated genetic differentiation between ADK genes in other species and upland cotton.

### 2.3. Chromosomal Mapping and Gene Duplication of the ADK Gene Family in G. hirsutum

**Figure 2:**
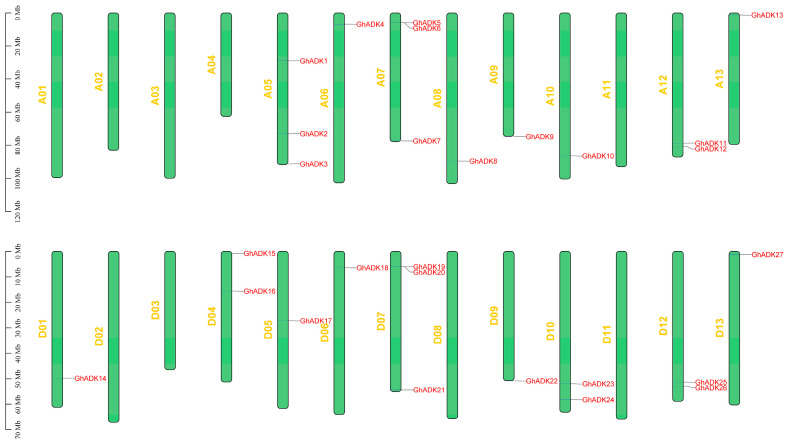
The chromosomal distribution of *ADK* genes in upland cotton (*Gossypium hirsutum*).

To further explore the molecular mechanisms underlying the expansion in *G. hirsutum*, we first mapped all identified *GhADKs* onto the chromosomes (Figure 2).

As a result of the analysis, we found that 27 of the 30 *ADK* genes were distributed on the chromosomes randomly, with the remaining 3 *ADK* members located on the scaffolds, which are not shown in Figure 2. Moreover, 13 *GhADKs* were located at the At-subgenome and 14 *GhADKs* were located at the Dt-subgenome. Among them, ten chromosomes contained only one member, while four chromosomes contained two genes, and three chromosomes contained three genes. Interestingly, most ADKs were distributed on both ends of the chromosomes. We also found that the numbers of genes on some chromosomes were not the same, such as for A01 and D01, A04 and D04, A05 and D05, A08 and D08, and A10 and D10, which may be caused by the incomplete genome-sequencing or the gene being lost during evolution. In particular, we did not identify tandem duplication events (TEDs) in *GhADKs*, which also occurred in G. arboretum, *G. raimondii*, and *G. barbadense*. 

**Figure 3:**
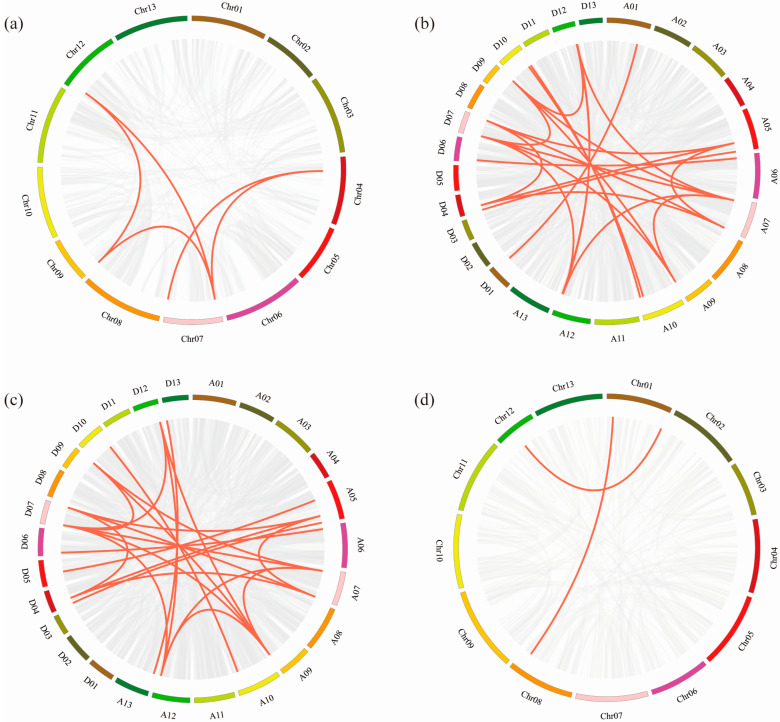
Schematic representation of the duplication of *ADK* genes in the cotton genome. The gene duplication events from (**a**) *G. arboretum*, (**b**) *G. barbadense*, (**c**) *G. hirsutum*, and (**d**) *G. raimondii* were exhibited on their respective chromosomes. The number of each chromosome is indicated inside each bar. The scale on the box above is in megabases (Mb). *ADK* gene pairs with a syntenic relationship are linked by red lines.

To better analyze the evolutionary relationship of the *ADK* gene family in *G. hirsutum*, we chose *G. hirsutum* as the main species and other cotton species as the control. The syntenic and collinearity maps were conducted on *ADK* genes of four cotton species (Figure 3).

As the result shows, both allotetraploid cotton species, *G. hirsutum* and *G. barbadense* (AD genome), contained 24 pairs of segmental duplication genes, respectively. In the diploid ancestral cotton species, *G. arboretum* only contained five pairs of segmental duplication genes, while *G. raimondii* contained the fewest, with two pairs. The results of the collinearity analysis revealed that the main expansion mechanisms of the cotton species *ADK* gene family are polyploidy and segmental duplication events.

**Figure 4:**
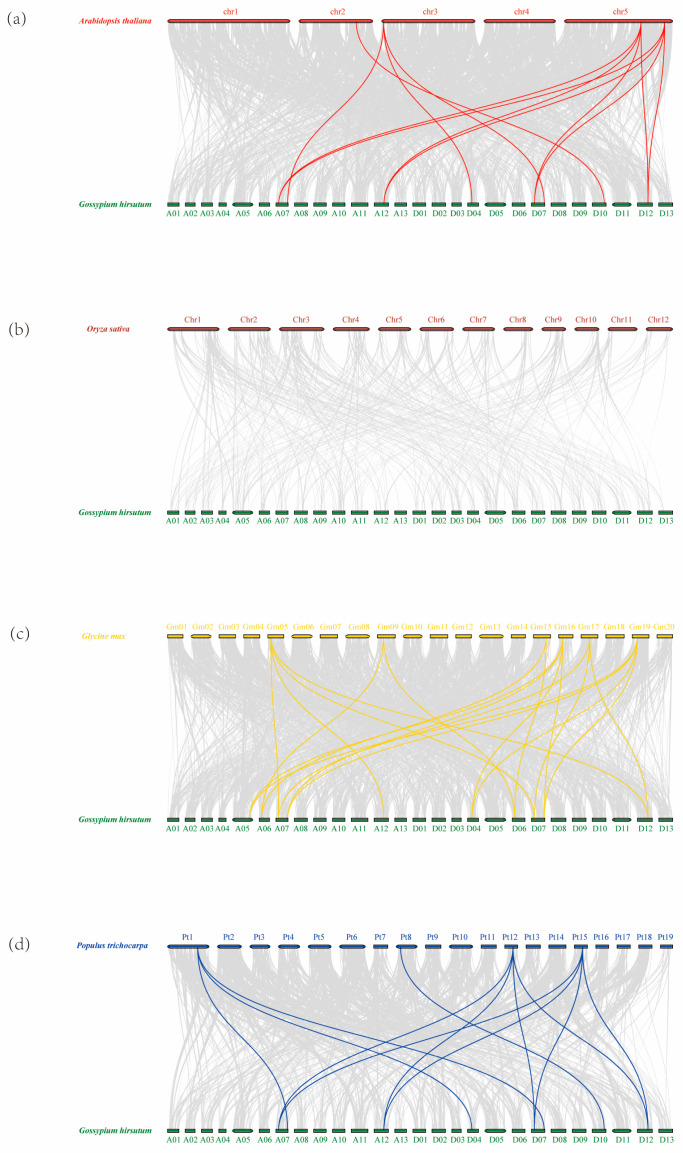
Synteny analyses of *ADK* genes between *G. hirsutum* and (**a**) *A. thaliana*; (**b**) *O. sativa*; (**c**) *Glycine max*; and (**d**) *Populus trichocarpa*.

**Figure 5:**
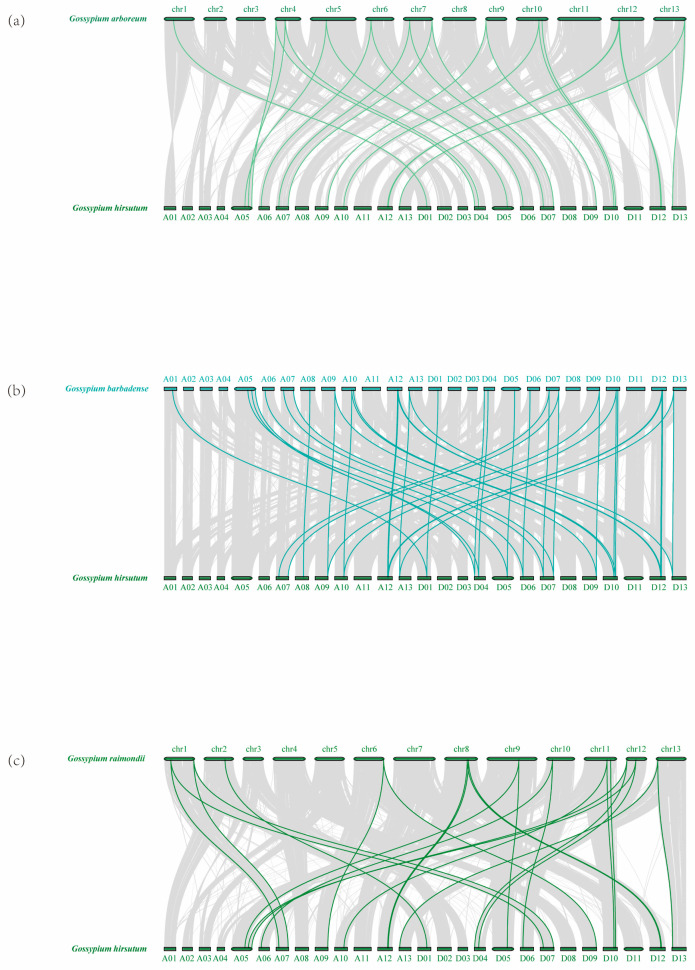
Synteny analysis of *ADK* family genes in cotton. Orthologous relationships of *ADK* genes were investigated between (**a**) *G. hirsutum* and *G. arboretum*; (**b**) *G. hirsutum* and *G. barbadense*; and (**c**) *G. hirsutum* and *G. raimondii*.

In addition, homology analyses of the *ADKs* between *G. hirsutum*, *G. arboretum*, *G. raimondii*, *G. barbadense*, Arabidopsis, Rice, Populus trichocarpa, and Glycine max were conducted. As shown in Figure 4 and Figure 5, the *ADK* genes in upland cotton had the most homologous gene pairs in *G. barbadense* (53 orthologous gene pairs), followed by *G. arboretum* (26 orthologous gene pairs) and *G. raimondii* (26 orthologous gene pairs). However, fewer homologous genepairs were observed between *G. hirsutum* and Arabidopsis (12 orthologous gene pairs), Rice (no orthologous gene pairs), Glycine max (19 orthologous gene pairs), and Populus trichocarpa (14 orthologous gene pairs).

### 2.4. Gene Structure Analysis and Conserved Motif Detection of Upland Cotton ADK Genes

**Figure 6:**
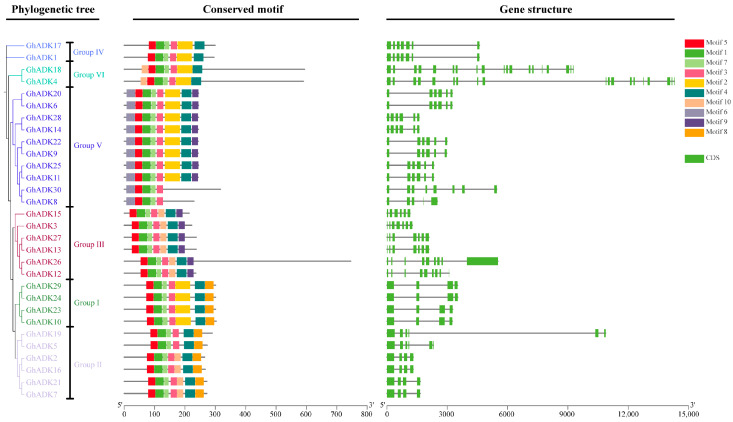
Conserved motifs and structure of *ADK* genes in upland cotton. Maximum likelihood phylogenetic tree of the *GhADKs*. The Conserved motifs in *ADK* genes were predicted, and the different colors represent different motifs. The intron–exon structures of the *ADK* genes were analyzed. The exons and introns were represented by yellow boxes and gray lines, respectively.

The gene structure can provide valuable information regarding possible evolutionary relationships and gene functions. Therefore, the organization of exons and introns in all *GhADK* genes was investigated through phylogenetic analysis (Figure 6).

This is consistent with the result in Figure 1. In the *GhADK* gene family, the number of exons varied from 4 to 19. The result showed that the members were clustered in the same subgroups and generally shared highly similar exon–intron structures, including intron numbers and exon/intron lengths. We also found that the length of exon s in the same subgroup was identical, while the intron length varied a lot. Our results also showed that *GhADK* members shared a similar genetic structure with some differences; for example, in Group V, most members contained six exons, but *GhADK30* contained eight exons, while GhADK8 contained five exons. This raises the possibility of a certain degree of functional diversity among the genes in this family.

To further characterize the structural diversity and determine the functions of *GhADKs*, the MEME suite software was used to identify 10 motifs among 30 members. As shown in Figure 5, motifs 1, 3, 5, and 7 were widely distributed in all *GhADKs*, which were annotated to encode the *ADK* domain based on a Pfamscan and SMART data search. However, some motifs were recognized to be specific to certain subgroups; for example, motif 6 was unique to Group V, motif 9 was unique to Group V and Group III, and motif 8 was only found in Groups I and II. Moreover, in Group V, *GhADK8* and *GhADK30* only contained five motifs, which were different from those of other members. Thus, we supported the idea that changes in gene function or errors in genome annotation may cause genes in the same family.

Substantially, genes in the same group shared a similar gene structure and motif organization, suggesting that these proteins may all have similar functions. However, differentiating group-specific gene structures and motifs would allow for functional specialization.

### 2.5. Analysis of Cis-Acting Elements of the G. hirsutum ADK Gene Promoter

**Figure 7:**
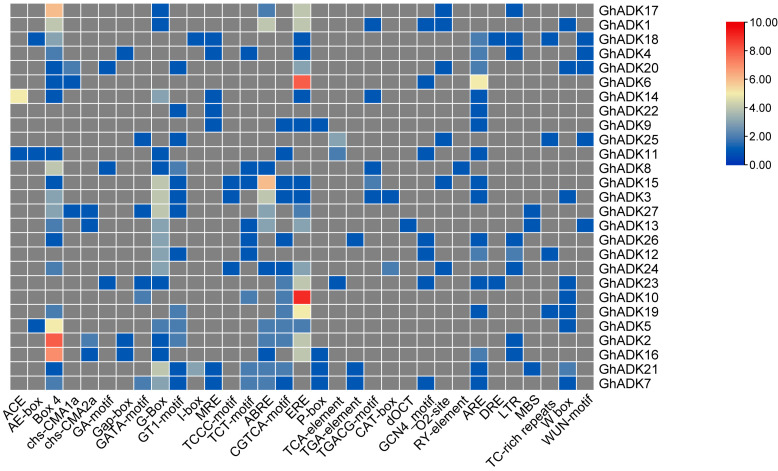
The distribution of cis-acting elements in promoters of *GhADKs*. The corresponding number of them was indicated by the color scale.

Cis-acting regulatory elements play a key role in the regulation of gene transcription initiation through interacting with their corresponding trans-regulatory factors, especially encountering environmental biotic and abiotic factors. To further study the transcriptional regulation mechanism of the *GhADK* gene family, the cis-acting elements in the upstream 2000 bp promoter sequences of 30 *GhADK* genes were analyzed (Figure 7).

Generally, these identified cis-elements were divided into four functional categories: light response, hormone response, development regulation, and stress response. The detailed information of putative cis-acting elements in each *GhADK* is listed in Additional File S5 (in this study, we only selected the + chain for the statistic). Among them, the promoters of 30 *ADK* genes contained at least one light-responsive element (e.g., Box 4, G-box, and GATA-motif), especially the two most abundant type elements, Box 4 and G-box. The upstream regions of most *ADK* genes contained a phytohormone-related component, such as ABRE, ERE, and P-box. Furthermore, we also identified cis-elements involved in plant growth and development, such as CAT-box, dOCT, and GCN4_motif. In addition, the promoters of 30 *ADK* genes contained one or more stress-related elements; ARE involved in the regulation of gene expression in the absence of oxygen was the most abundant, followed by MBS (MYB binding site involved in drought-inducibility), LTR (involved in low-temperature responsiveness), and DRE involved in dehydration, low temperatures, salt stresses, and so on. These findings suggested that *GhADKs* may be in various stress responses, which can provide more helpful information in exploring the regulatory mechanisms of the *G. hirsutum ADK* gene family.

### 2.6. Expression Profiles of GhADK Genes under Cold Treatment and Drought Stress

Previous studies have shown that *ADK* genes regulate plant responses to abiotic stressors (drought and cold). To investigate the possible functions of the ADK gene family in *G. hirsutum*, the expression profiles of ADK genes exposed to low-temperature treatment (4 °C) and drought treatment were also examined based on transcriptome data. We analyzed the expression patterns of the upland cotton *GhADKs* gene after 0, 1, 3, 6, 12, and 24h of cold or drought treatment, respectively. *GhADK6/22/23/24/29* could not be detected in the two stresses. We deducted these genes as pseudogenes, or they may be expressed only under particular conditions.

**Figure 8:**
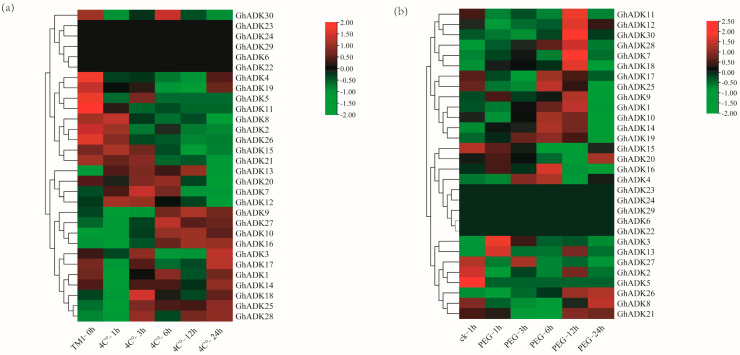
Expression analysis of *GhADK* genes in *G. hirsutum* under cold and drought stresses. The RNA-Seq expression profiles of *G. hirsutum* were used to identify the relative expression levels of *GhADK* genes. Levels of gene expression are depicted in different colors on the scale. cold (**a**) and drought (**b**) treatments, the red color represents a high expression and the green color represents a low expression. The detailed FPKM values are present in Additional File S6.

The results indicated that, under cold stress, the majority of the *GhADK* genes (25 out of 30) were consistently induced during the treatment (Figure 8a).

Among them, six genes were upregulated and nineteen genes were downregulated at 1 h, suggesting that these genes were regulated for a rapid response to cold stress. *GhADK17/18/19/21/25/28* showed a down–up–downregulation trend under cold treatment. Two genes (*GhADK18/28*) reached their peak expression at 3 h, and *GhADK1/GhADK20* reached their peak at 6 h, indicating that these genes may be cold acclimation genes. After 12 h of treatment, *GhADK4* and *GhADK19* were significantly down-regulated and *GhADK13/16/25* were upregulated considerably, suggesting that these genes may be involved in a cascade of downstream signal pathways by interacting with other proteins. Also, *GhADK4/8/21/30* were significantly downregulated and *GhADK16/25* were significantly upregulated after 24 h of cold treatment; these genes could be crucial for long-term cold acclimation. In these cold-induced genes, *GhADK19*, *GhADK20*, and *GhADK25* were highly expressed at every treatment point; contrarily, most genes had low expression at all times. These fundings suggest that this gene family may play a negative regulatory role in cold stress responses.

During drought stress, *GhADK3* was significantly upregulated at the 1 h point and dramatically downregulated at 3/6/12/24h. GhADK8/15/21/27 were down-regulated at 6 h, while *GhADK28* was significantly upregulated. Among the *GhADKs*, *GhADK1/9/12/18/21/28/30* reached their peak expression at 12 h, and *GhADK8/20/26* reached their peak expression at the 24 h time point. Four genes (*GhADK19/20/25/28*) were highly expressed at every moment; contrarily, most genes have been at relatively low expression levels. Combining the transcriptomic data under cold and drought conditions, we found that *GhADK7/13* were upregulated and 12 *GhADKs* were downregulated at 1 h. Seven genes were upregulated and eighteen were downregulated at 3 h under cold and drought conditions. These results suggested that each *ADK* member had its unique inducible expression pattern and thus may have its own functional specificity in plant stress responses.

### 2.7. qRT-PCR Verification of Upland Cotton ADK Family Members

**Figure 9:**
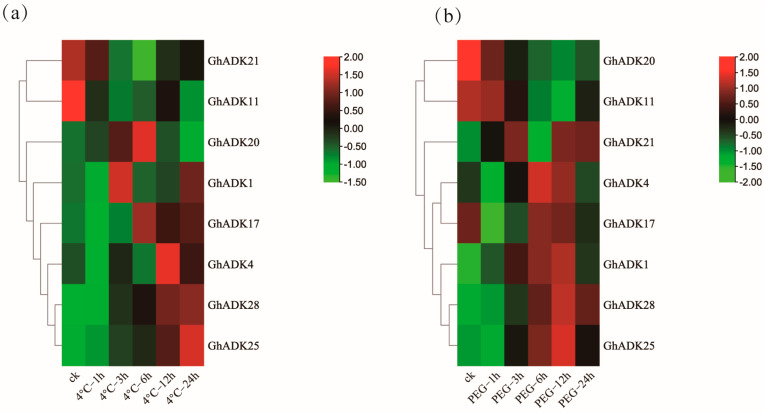
Through qRT-PCR analysis, the expression profiles of *GhADKs* under various stress treatments were studied. Treated cotton leaves were harvested at the specified time points. To ensure that the expression of the same gene under different treatments as well as the expression of different genes under the same treatment can both be clearly displayed and compared for cold (**a**) and drought (**b**) treatments, the color scale represents the log2 mean value of relative expression levels from three independent biological replicates (*n* = 3).

Transcriptome data analysis showed that most *GhADK* members exhibited different expression patterns under various stress treatments. To verify the reliability of the above results, several candidate genes with altered expression levels were randomly selected for qRT-PCR experiments. The treated cotton leaves were harvested at the indicated time points. Primers specific to *GhADK* were used, with the details listed in Supplementary Materials Table S3. The expression patterns of eight genes (*GhADK1*, *GhADK4*, *GhADK11*, *GhADK17*, *GhADK20*, *GhADK21*, *GhADK25*, and *GhADK28*) were examined after 0, 1, 3, 6, 12, and 24 h of cold or drought treatment (Figure 9).

*GhADK1*, *GhADK4*, *GhADK17*, *GhADK21*, *GhADK25*, and *GhADK28* showed an upregulation trend under cold stress, with elevated expression levels in the later stages. *GhADK1* was significantly upregulated at 3 h, indicating a rapid response to cold stress. *GhADK20* and *GhADK17* were significantly upregulated at 6 h. *GhADK1* was significantly downregulated at 12 h. *GhADK4*, *GhADK17*, *GhADK25*, and *GhADK28* had higher expression levels at 12 and 24 h, suggesting these genes may be crucial for long-term cold adaptation. Under drought stress, *GhADK11* and *GhADK20* were upregulated at 1 h and downregulated at 6, 12, and 24 h. Among the *GhADKs*, *GhADK1*, *GhADK4*, *GhADK17*, *GhADK21*, *GhADK28*, and *GhADK25* reached their peak expression at 12 h.

Combining the transcriptome data under cold and drought conditions, we found that *GhADK21* was upregulated at 1 h, while 5 *GhADKs* were downregulated at 1 h. At 12 h, four genes were upregulated and four genes were downregulated. These results suggest that each *ADK* member has its unique inducible expression pattern and may therefore have its own functional specificity in plant stress responses.

### 2.8. Functional Analysis of GhADK25 Silencing in Cotton under Drought and Cold Stress

**Figure 10:**
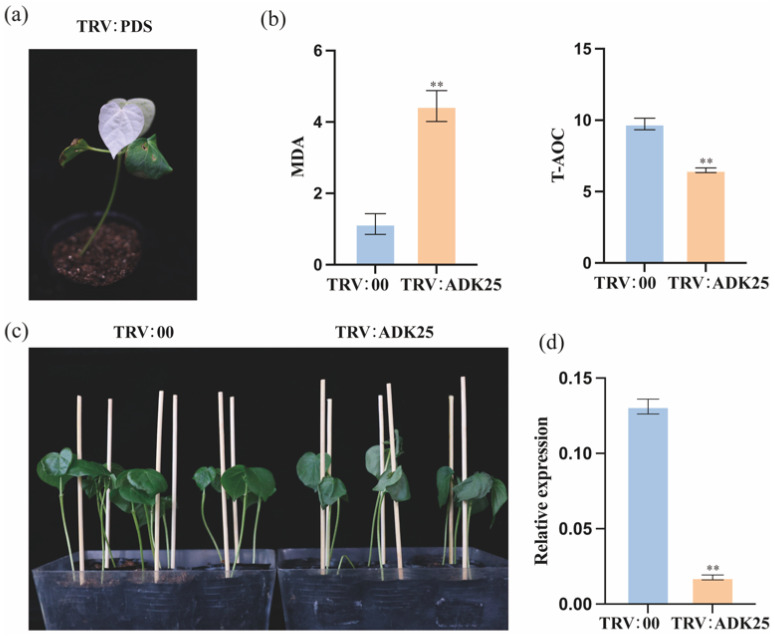
Regulation of ADK25 under cold stress. (**a**) Albino phenotype. (**b**) Effects of silencing ADK25 on the MDA content and T-AOC in cotton under cold stress. (**c**) Phenotype of plants; negative control (**left**) and silenced plant (**right**). (**d**) Silencing efficiency of ADK25 was detected by qRT-PCR. Asterisks represent differences by Student’s *t*-test: **, *p* < 0.01 (*n* = 3).

**Figure 11:**
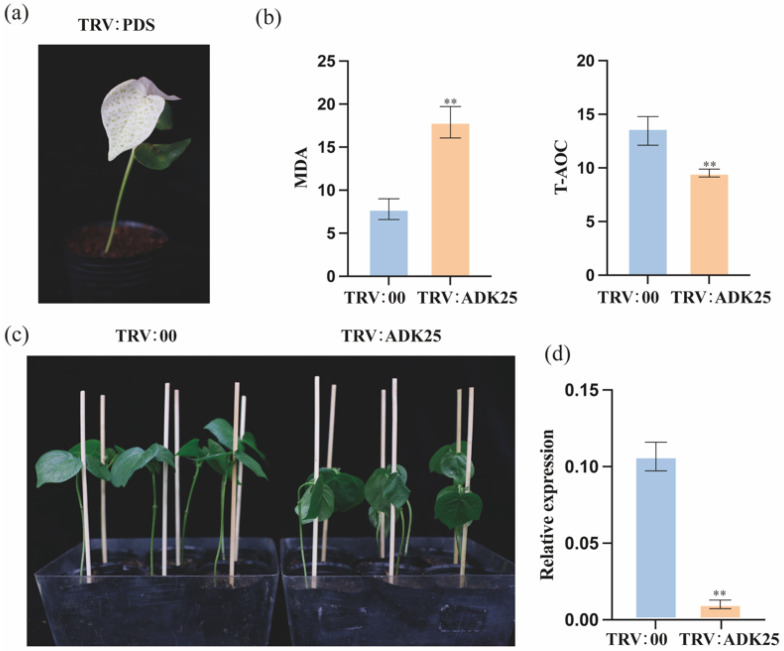
Regulation of ADK25 under drought stress. (**a**) Albino phenotype. (**b**) Effects of silencing ADK25 on the MDA content and T-AOC in cotton under drought stress. (**c**) Phenotype of plants; negative control (**left**) and silenced plant (**right**). (**d**) Silencing efficiency of ADK25 was detected by qRT-PCR. Asterisks represent differences by Student’s *t*-test:; **, *p* < 0.01 (*n* = 3).

Based on the results of qRT-PCR, a candidate gene *GhADK25* was selected for VIGS gene silencing. Injection was performed on two cotyledons of the test variety TM-1 and after 7 days of treatment, as shown in cold (Figure 10a) and drought (Figure 11a).

The appearance of bleaching in the cotyledons indicated successful PDS silencing. Subsequently, samples were taken from the remaining silenced plants, with each sample subjected to qRT-PCR validation in triplicate and compared with control groups and untreated materials. The results revealed that the control group TRV:00 (referred to hereafter as the control group) exhibited significantly better leaf and plant growth under cold and drought stress compared to silenced plants (Figure 10c and Figure 11c). The qRT-PCR silencing efficiency detection results are shown in Figure 10d and Figure 11d. Compared to the control group, the expression level of *GhADK25* in the silenced plants was significantly reduced, indicating that the viral injection caused the differential expression of GhADK25 in the silenced plants. In addition, the physiological and chemical characteristics of MDA (malondialdehyde) and T-AOC (total antioxidant capacity) showed significant differences between *GhADK25*-silenced plants and controls. Compared to the controls, the T-AOC in the silenced seedlings decreased, while the MDA increased. These results indicate that silencing *GhADK25* compromises the plant’s ability to tolerate drought and cold stress (Figure 10b and Figure 11b).

## 3. Discussion

Cotton (*Gossypium* spp.) is one of the most important economic crops worldwide as a source of natural fibers, edible oil, and protein. With the continuous development of sequencing technology, the genomes of cotton species have been sequenced, already making the genome-wide identification and analysis of gene families in plants common. ADK proteins were reported to be involved in the regulation of plant growth and development and in responses to various environmental stresses in plants [13]. However, systematic studies of *ADK* genes in cotton species are lacking. Hence, our research conducted a comprehensive exploration of the *ADK* family in cotton, mainly focusing on the allotetraploid cotton *G. hirsutum*, to understand its roles, their evolutionary relationships, and its expression in response to abiotic stresses.

### 3.1. Systematical and Comprehensive Analyses of ADK Proteins

In the present study, we identified 16, 14, 30, and 32 ADK proteins in *G. arboreum*, *G. raimondii*, *G. hirsutum*, and *G. barbadense*, respectively. Our results showed that the number of ADK proteins in tetraploid cotton is almost twice as much as that in diploid cotton, which indicates that allotetraploid cotton is produced via the natural hybridization of diploid cotton seeds containing the A genome and D genome [22]. The phylogenetic tree suggested that ADK proteins in cotton could be divided into six groups, similar to Arabidopsis and other plants. Each subgroup contains the *ADK* genes of four cotton species; the number of ADK proteins in some branches differed from that of different species. For example, group V included 10, 6, 10, and 4 *ADK* genes of *G. hirsutum*, *G. arboreum*, *G. barbadense*, and *G. raimondii*, respectively, but only 2 in arabidopsis, 3 in rice, 2 in potato, and 2 in tomato. This situation is also seen in other groups. ADK proteins were unevenly distributed on 16 chromosomes in upland cotton, but we also found that the number and location of genes on each chromosome were almost one to one. The gene’s structure and motif analyses indicated that the *ADK* genes in the same subgroup shared similar arrangements, increasing the gene diversity among different subgroups. Notably, in the same subgroup, *GhADK8* and *GhADK30* lacked motifs 2, 4, and 9, and *GhADK4* and *GhADK19* have a long intron, which made them different from other *ADK* gene members because of their large size, these gains or losses suggesting that these motifs and different structures might be involved in the functional divergence of *GhADKs*. The analysis of cis-acting elements of the *G. hirsutum ADK* gene promoter showed that the *G. hirsutum ADK* family may exert its biological functions mainly through different pathways.

### 3.2. Evolution and Expansion of the ADK Gene Family in Cotton

To date, *ADK* genes have been identified in various plants. The number of these *ADKs* in Gossypium was more significant than that in other species such as arabidopsis (7), rice (7), potato (12), and tomato (11) [10,13,23], indicating that more ADK proteins in cotton participate in the stress-resistant signaling pathway. Generally, the size of the gene number may be affected by two reasons: the genome sizes and the duplication event. For example, the upland cotton has a greater than 2.27 GB genome [24], while Arabidopsis only contains a 125 Mb genome, rice only contains a 386 Mb genome, tomato contains a less than 1.2 Gb genome, and so on [25,26,27]. Next, homologous exchanges were present in the upland cotton genome, and *G. raimondii* underwent a whole-genome duplication event about 13–20 million years ago [28].

Tandem duplication and segmental duplication events are the main driving forces of gene family expansion, which can help plants adapt to varying environmental conditions [29]. A total of 24 putative segmental gene pairs with 22 upland cotton *ADK* genes were identified in the whole genome. This finding suggests that more than half of the *ADK* genes in *G. hirsutum* may not originate from the same ancestor. We did not find a tandem duplication event in four cotton genomes. A similar phenomenon was observed in the tomato *ADK* gene family: it contained only three pairs of duplicated genes among a total of 11 tomato *ADK* genes [23]. According to these results, we supposed that the expansion of the *ADK* gene family may be mainly through the segmental supplication event. On the other hand, the number of *ADK* genes in the two tetraploid species was almost the sum of those in the two ancestral diploid progenitors, which provided evidence that polyploidy is another important factor. At the same time, we found that the ratio of *ADKs* in allotetraploid cotton and diploid cotton was less than 2:1, which may be the result of evolutionary selection in the process of hybridizing two diploid cotton plants to form allotetraploid cotton.

All the duplicated genes in cotton underwent the purifying selection, as indicated by the Ka/Ks ratio being <1, suggesting that all of those duplicated genes experienced a solid purifying selective pressure and subfunctionalized during evolution.

### 3.3. Gene Expression Profiles and Functional Divergence of ADKs in G. hirsutum

Expression analyses could provide insight into the potential functions of genes. In many plants, the *ADK* genes responded rapidly to biotic and abiotic stresses [15,18]. Hence, the expression pattern of *GhADK* genes under cold and drought conditions was performed using publicly available RNA-seq data. Interestingly, most duplicated genes showed different expressions between the duplicates. For example, *GhADK11* and its paralog *GhADK20*, *GhADK11* and its paralog *GhADK9*, and *GhADK11* and its paralog *GhADK25* displayed distinct expression patterns under cold stress. These findings indicate different roles of these genes under various stress. However, some duplicated genes still showed a similar expression between the duplicates. For example, *GhADK9* and its paralog *GhADK25*, *GhADK28* and its paralog *GhADK14*, and *GhADK1* and its paralog *GhADK17* showed the same expression patterns under cold stress. This is also the case under the drought treatment; for example, *GhADK9* and its paralog *GhADK25* and *GhADK13* and its paralog *GhADK27* showed the same expression patterns. These results suggest that they might have redundant roles. All these findings suggest that the duplicated genes might undergo different evolutionary pressures, which helped in analyzing the functional genes in upland cotton.

Also, we found that most *GhADKs* presented opposite expression patterns under cold and drought stress. For example, *GhADK8*, *GhADK21*, and *GhADK26* were downregulated during cold stress but were upregulated under drought stress. *GhADK27* and *GhADK3* were upregulated during cold stress but were downregulated under drought stress. This phenomenon is similar to the expression pattern of *ADK* members in Medicago sativa. In tomato studies, it was shown that most *SlADK* genes were upregulated by drought induction [23]. In upland cotton, the situation was similar. So, the expression patterns of *ADK* genes show that there are differences between different species under abiotic stress, suggesting that positive and negative regulatory mechanisms may exist.

### 3.4. The Potential Roles of ADK Genes in Response to Cold and Drought Stress

In plants, adapting to abiotic stresses such as cold and drought is crucial for survival and productivity. Adenylate kinase (*ADK*) genes have been implicated in regulating plant responses to these stressors, particularly energy transfer and metabolic homeostasis. Under 4 °C treatment, *GhADK25* was induced between 3 and 24 h (Figure 9b). Further functional analysis of *GhADK25* using the VIGS method showed that under cold conditions, plants with silenced *GhADK25* suffered more damage compared to control plants (Figure 10c). In our study, *GhADK25* was also induced between 3 and 24 h after PEG 6000 treatment (Figure 9a). Further functional analysis using VIGS revealed that under drought conditions, plants with silenced *GhADK25* suffered more damage than the control plants (Figure 11c). Understanding the potential roles of ADK genes in cold and drought stress tolerance can provide valuable insights into the molecular mechanisms underlying plant stress adaptation.

Previous studies, including those referenced in the material text, have demonstrated that *ADK* genes play a pivotal role in regulating the energy transfer rate and effectiveness from mitochondria to hexokinase in vitro [1]. This function is critical for maintaining metabolic homeostasis under stressful conditions, where energy production and utilization are often compromised. *ADK* enzymes catalyze the reversible phosphorylation of AMP and ADP, which is a crucial step in maintaining the adenylate pool and energy charge [30]. By modulating the levels of AMP, ADP, and ATP, *ADK* genes can influence the cell’s metabolic state and its ability to respond to stress.

## 4. Materials and Methods

### 4.1. Identification and Bioinformatics Analysis of the ADK Gene Family in Cotton

The genome datasets of *Gossypium arboreum*, *Gossypium raimondii*, *Gossypium hirsutum*, and *Gossypium barbadense* were downloaded from the COTTONGEN (https://www.cottongen.org/ (accessed on 3 April 2024)) [31] database. Two methods were used to identify cotton ADK family genes comprehensively. First, the ADK protein sequences of Arabidopsis, rice, and soybean were obtained from the Uniprot (https://www.uniprot.org/ (accessed on 3 April 2024)) [32] website and were used as query sequences to conduct a local BLASTP analysis against the cotton genomic database, with an e-value of 1 × 10^−50^ as the threshold. Second, the hidden Markov model (HMM) file corresponding to the ADK domain (PF00406) was downloaded from the Pfamscan database (https://www.ebi.ac.uk/Tools/pfa/pfamscan/ (accessed on 3 April 2024)) [33]; then, the HMM model was used as a probe to perform a BLASTP against the cotton genome database under the bio-Linux operating system, with the threshold expectation value set to 1 × 10^−20^. Then, the standard IDs of the genes obtained by the two methods were selected as the candidate genes. The conserved domains of the candidate sequences were annotated using the SMART (http://smart.embl-heidelberg.de/ (accessed on 3 April 2024)) [34], Pfamscan, and CDD Search (https://www.ncbi.nlm.nih.gov/Structure/cdd/wrpsb.cgi (accessed on 3 April 2024)) [35] for detecting the *ADK* domain. The biophysical properties of potential *GhADKs*, including the number of amino acids, molecular weights (MW), and isoelectric points (pI), were generated using ExPASy (http://web.expasy.org/protparam/ (accessed on 4 April 2024)) [36]. The subcellular localization of the ADK proteins were predicted via the online website Softberry (http://www.softberry.com/ (accessed on 3 April 2024)) [37].

### 4.2. Multiple Alignments and Phylogenetic Analysis

The ADK protein sequences of Arabidopsis, rice, soybean, *Gossypium arboreum*, *Gossypium raimondii*, *Gossypium hirsutum*, and *Gossypium barbadense* were used for multiple sequence alignment using MAFFT version 7 (https://mafft.cbrc.jp/alignment/software/ (accessed on 7 April 2024)) [38] with auto strategy parameters. An unrooted phylogenetic tree was generated through IQ-TREE version 1.6.2 [39] with the Maximum Likelihood (ML) method. Branch support for the tree topology was estimated by using a bootstrap analysis with 1000 replicates. The resulting phylogenetic tree was visualized using the iTOL (http://itol.embl.de/help.cgi (accessed on 10 April 2024)) [40] online tool.

### 4.3. Chromosomal Localization and Gene Duplication Analysis

The chromosomal distribution of *GhADKs* genes was visualized by the MapInspect version 1.0 (https://mapinspect.software.informer.com/ (accessed on 3 April 2024)) graphical tool according to the genomic coordinates retrieved from the *Gossypium hirsutum* genome database. As previously studies described, gene duplication events included tandem and segmental duplication. Two or more genes located on the same chromosome were arranged at a 200 kb distance and shared more than 70% of their identities, as analyzed with BLASTP, which can be defined as tandem duplications. The Multiple collinear scanning toolkits (MCScanX) [41] with default parameters were used to detect the segmental duplication patterns of the *GhADKs*. The Circos program was used to draw collinearity maps of the duplicated gene pairs between four cotton species as well as the synteny blocks of the orthologous *ADK* genes between *Gossypium hirsutum* and Arabidopsis, soybean, poplar, and rice. To further analyze the divergence of duplicated genes, the synonymous substitution rate (Ks) and non-synonymous substitution rate (Ka) were calculated using the TBTOOLS [42] software (V2.097). Based on a rate of λ (6.1 × 10^−9^) substitutions per site per year, the divergence time (T) was calculated using the Ks value and the following formula: T = Ks/(2 × 6.1 × 10^−9^)10^−6^ Mya.

### 4.4. Gene Structure Analysis and Conserved Motif Detection of the ADK Gene Family in Gossypium hirsutum

To further study the gene structures of *GhADKs*, the coding sequence (CDS) and gff3 format files of *Gossypium hirsutum* were downloaded from the cotton genome database. Subsequently, the diagrammatic sketch containing CDS, UTR, and Intron was utilized by TBtools software. To investigate the conserved motifs of ADK proteins, the complete amino acid sequences were analyzed using MEME (Multiple Expectation Maximization for Motif Elicitation) (http://meme.nbcr.net/meme/cgibin/meme.cg (accessed on 12 April 2024)) [43]. The parameters were shown follows: the number of repetitions was set to zero or one; the maximum number of motifs was set to 15; and other optional parameters were set to default.

### 4.5. Promoter Cis-Regulatory Analysis of the ADK Gene Family in Gossypium hirsutum

To further investigate the cis-elements in the promoter, we obtained the 2 kbp promoter sequences of *GhADK* genes from the genome annotation files. Then, the obtained sequences were subjected to analysis using the PlantCARE Database (https://bioinformatics.psb.ugent.be/webtools/plantcare/html/ (accessed on 14 April 2024)) [44]. In this study, we only selected thosethat may be typical and functional cis-elements; the ubiquitous cis-acting elements in most genes were filtered out, such as CAAT-box, TATA-box, TATC-box, and so on. Also, Promoter sequences with three members (*GhADK28*, *GhADK29*, *GhADK30*) were not extracted and were therefore not analyzed.

### 4.6. Expression Analysis of GhADKs

The gene expression data of *GhADKs* under stress conditions such as cold, heat, drought, and salinity were obtained from unpublic transcriptome data. The transcript abundance was represented by fragments per kilobase of exon per million mapped reads (FPKM) values calculated based on RNA-Seq reads. To clearly see the difference in the expression level and more convenient statistics, the RPKM values were log2-transformed. The results were presented as heatmaps using TBTOOLS software (V2.097).

### 4.7. Plant Materials and Treatments, RNA Isolation, and Quantitative Real-Time PCR Analysis

Quantitative real-time PCR (qPCR) analysis: cDNA was synthesized from the isolated RNA using a reverse transcription kit. qPCR was performed using specific primers targeting the genes of interest and a SYBR Green-based master mix. The reaction mixtures were subjected to thermal cycling conditions, including an initial denaturation step, followed by a series of amplification cycles. The fluorescence signals were monitored during each cycle to quantify the accumulation of PCR products. The relative gene expression levels were calculated using the comparative Ct method, with normalization to a reference gene. Statistical analysis was performed to determine significant differences in gene expression between the treatment groups.

### 4.8. Virus-Induced Gene Silencing (VIGS) Treatment of ADK25 in Cotton Seedlings

Based on the gene silencing sequence design website (https://vigs.solgenomics.net/ (accessed on 3 April 2024) [22], a 300 bp specific coding sequence of ADK25 was designed for virus-induced gene silencing (VIGS) treatment. The primers used are listed in Additional File S7. The fragment was amplified and cloned into a tobacco rattle virus vector (pTRV2) using a ClonExpress^®^ Ultra One Step Cloning Kit (C115, Vazyme, Nanjing, China). TRV2::ADK25 was transferred into the Agrobacterium tumefaciens strain LGV3101. TRV2::00 was used as a negative control. The Agrobacterium tumefaciens strains of the vector were mixed with the Agrobacterium tumefaciens strains containing pTRV1 at a ratio of 1:1. The mixture was injected into the cotyledons of cotton seedlings using a 1 mL needleless syringe. After 24 h in the dark, the plants were cultivated in a constant temperature light room (28 °C, 16 h/8 h, day/night).

### 4.9. Measurement of Physio-Biochemical Attributes

Ion permeability was determined using 10 rosette leaves of identical sizes. Before and after treatment, the malondialdehyde (MDA) and total antioxidant capacity (T-AOC) were measured according to the manufacturer’s instructions (Solarbio, Beijing, China).

## 5. Conclusions

This study systematically analyzed the *ADK* gene family in cotton (*Gossypium hirsutum*) using bioinformatics methods. The results revealed significant variations in the length and molecular weight of GhADKs proteins, with a diverse range of theoretical isoelectric points, highlighting their structural diversity and potential functional differences. Subcellular localization predictions indicated that ADK proteins predominantly localize to the cytoplasm, mitochondria, and chloroplasts, suggesting specific functions and regulatory roles in different cellular compartments. Further transcriptome analysis unveiled dynamic expression patterns of the *ADK* gene family in response to abiotic stresses such as drought and cold in cotton. Particularly, the functional validation of *GhADK25* under drought stress using VIGS technology emphasized the crucial role of *ADK* genes in cotton’s response to environmental stresses. In summary, this study provides important foundational data and insights into the molecular mechanisms of *ADK* genes in cotton growth, development, and stress responses. Future research could explore the regulatory networks of the *ADK* gene family under different growth stages and environmental conditions, as well as their potential applications in breeding and improvement efforts.

## Figures and Tables

**Table 1:** The information of *ADK* gene family in *G. hirsutum*.

Gene ID	Gene name	Chr	Start	End	PI	MW (kDa)	AA
GhADK1	Gh_A05G2360	A05	28948631	28953225	7.14	32528.07	296
GhADK2	Gh_A05G2963	A05	73057726	73059034	6.15	29723.98	265
GhADK3	Gh_A05G3557	A05	91270041	91271305	6.37	25040.75	222
GhADK4	Gh_A06G0410	A06	6886337	6900641	8.56	65724.32	590
GhADK5	Gh_A07G0462	A07	5972595	5974906	7.11	30968.44	273
GhADK6	Gh_A07G0465	A07	5995641	5998894	6.97	26899.23	245
GhADK7	Gh_A07G2086	A07	77433461	77435110	6.15	30273.43	272
GhADK8	Gh_A08G1415	A08	89538235	89540746	8.5	24830.9	230
GhADK9	Gh_A09G2184	A09	74822698	74825672	7.64	26854.17	244
GhADK10	Gh_A10G1601	A10	86412249	86415506	6.37	32856.6	303
GhADK11	Gh_A12G1706	A12	78779245	78781576	7.69	26803.97	244
GhADK12	Gh_A12G1810	A12	80690562	80693656	8.49	26316.29	236
GhADK13	Gh_A13G0108	A13	1268419	1270512	7.03	26556.53	237
GhADK14	Gh_D01G1585	D01	49745145	49746750	6.91	26725.94	245
GhADK15	Gh_D04G0048	D04	774522	775676	7.6	24074.71	214
GhADK16	Gh_D04G0753	D04	15612652	15613961	6.15	29856.14	267
GhADK17	Gh_D05G2627	D05	27184500	27189101	8.41	32934.66	299
GhADK18	Gh_D06G0443	D06	6370844	6380120	8.94	66355.03	594
GhADK19	Gh_D07G0526	D07	5948360	5959238	9.08	33159.95	290
GhADK20	Gh_D07G0529	D07	5978760	5982018	6.55	26957.26	245
GhADK21	Gh_D07G2304	D07	54335943	54337596	6.51	30197.38	272
GhADK22	Gh_D09G2390	D09	50793345	50796339	8.22	26720.04	244
GhADK23	Gh_D10G1856	D10	51921697	51924967	6.24	32658.46	301
GhADK24	Gh_D10G2125	D10	58219589	58223112	8.37	32771.56	301
GhADK25	Gh_D12G1868	D12	51286089	51288425	8.26	26892.19	245
GhADK26	Gh_D12G1980	D12	52858886	52864409	8.52	83784.67	746
GhADK27	Gh_D13G0125	D13	1246139	1248231	8.4	26423.47	237
GhADK28	Gh_A01G2126	scaffold147_A01	199905	201512	6.96	26852.05	245
GhADK29	Gh_A10G2351	scaffold2716_A10	5914	9425	8.37	32710.48	301
GhADK30	Gh_A12G2555	scaffold3185_A12	1180857	1186323	9.68	34073.49	317

## Data Availability

Data is contained within the article or Supplementary Material.

## Supplementary Materials

The following supporting information can be downloaded at: https://www.mdpi.com/article/10.3390/ijms25147821/s1.
