# Supplementary material for: Genome-Wide Identification and Expression Analysis of ADK Gene Family Members in Cotton under Abiotic Stress
Source: Int J Mol Sci. 2024 Jul 17;25(14):7821. doi: 10.3390/ijms25147821 (PMC11277214; doi:10.3390/ijms25147821)
Supplement: Supplementary file 1 [file ijms-25-07821-s001.zip › Additional file S1.pdf]

The protein sequences of the ADK proteins from cotton species and *A. thaliana, O. sativa, S. lycopersicum & S. tuberosum*.

G. arboretum

>GaADK1

MADSAVALEDVASVDIMTELLRRFKCSSKPDKRLILIGPPGSGKGTQSPVIKDDYCLCHLATGDMLRA
AVAAKTPLGIKAKEAMDKGELVSDDLVVGIIDEAMKKTSCQKGFILDGFPRTVVQAQKLDEMLEKQG
TKIDKVLNFAIDDSILEERITGRWIHPSSGRTYHTKFAPPKVPGLDDVTGEPLIQRKDDTPAVLKSRLEAF
HRQTEPVIDYYGSKGILANLPAEKPPKEVTSEVQKVLSS

>GaADK2

MGTANDSSKDISDRLAEKHPKVVFVLGGPGSGKGTQCEKIVQHFGYTHLSAGDLLRAEKNSGSENGI
MIQNMMNEGKIVPSEVTIKLLQKAMLDSGNDKFLIDGFPRNEENRAAFEALTKIEPEFVLYFNCPEKEM
ERRILNRNQGREDDNIETIRKRFKVFLESSLPVIEYYKGKGKVREIDAAKPVEEVFEEVKAIFNPKVPKVPK
DENVEGRCVCCAIL

>GaADK3

MAWLPKLRVASASVASSAVNPLRRWIRDIGSAAALELDYDYDYYEYERAPRDRRLPQQKLDVDGSVP
ERGVQWVLIGEPGVKRHAYAERLSKLLGIPHISMGSLIRQELDPYSSLYKQVVNAVNEGKLVPEDVVFA
LLSKRLEEGENGFILDGIPRTRLQAEILDQIVDIDLVVNFKCSEEYMKMSSESELLHIGNSKGVESSLKKLR
VYSEQAKSVEDYYSKQKKLLNFQLAGATGDAWQGLLAALHLQYSPINALSSSPKLTA

>GaADK4

MGSTSFSPSISLSPSLSPHSSKFPNPSPNLFLRSHSSFTPLTLRSNQTRYRFKKLPSSNAPNFLVAASAKA
EPLKVIISGAPASGKGTQCELITQKYGLVHIAAGDLLRAEVAAASENGKLAKEYMEKGELVPDEIVVMM
VKERLLQPDSQQRGWLLDGYPRSSSQAAALEDYGIRPDVFILLDVSEDILVERVVGRRLDPLTGKIYHL
KYSPPENDEIASRLTQRFDDTEEKVKLRLRTHHQNVDAVLSVYKDITVKVNGNEAKEHVFAQIDAALT
HVGEQRKVNSGSVAA

>GaADK5

MLATSSSSSSSTAISLHPLANYNASATPSLNPSSLSFSSSYSYLSPLSFRLHSSKTHLRITPKKGLRVSCST
NEPLKVMISGAPASGKGTQCELIVQKFGLVHISTGDLLRAEVSSGTEIGNKAKEFMNSGRLVPDEIVTA
MVTVRLSRQDAKEKGWLLDGYPRSFAQAQSLEELNIRPDIYIVLDVPDEILIDRCVGRRLDPVTGKIYHL
KNFPPESEEIKARLVTRADDTEEKVKSRLEIYKQNAAAISSTYSSITNKIDGNRPKEIIFKDIDSLLSQLLKD
KTAKSVKPVLQTENCLDQASSNEEKWRGIPTRLNNIPHSREIRNYFYDDVLQATQRAVNDGRTRLKV
EINIPELNPEMDVYRIGTLMELVRVIALSFADDGKRVKVCVQGSMGEGALAGMPLQLAGTRKILEFMD
WGNYGAMGTFIKIGSIGAKEVDEEDDMFILVAPQNAVGNCIIDDLRAMTDAAGKRPVILINPRLKDLP
GSSGIMQTMGRDKRLEYAASFESCYFFRLLYYAGTQYPIMGAIRMTYPYDYELYRRVDEPSGKEKYVSL
SIFKERPTIDEINDAFLGKPRNKDKKASGIWGFLSGVF

>GaADK6

MIINKLSHLATVTTATTAAVTAAPPLYRLLKLTDSLFHGAAQPQPDTDYWYYYPPSQQESRRDELVRS
TPVADTDGSVPLRGVQWAFIGSPRAKKRVYAVMLSKLLEVPHITMASLVRQELSPNSNLYKQIANAV
DHGELVNEDIILGLLSKRLEDGHYRGETGFILDGIPRSRTQAEILDQLAEIDLVVNFKCTEDLMMNNQG
EASWTERLQDYIKQSKVVEEYYKNQKKLLEFQIGNASMETWRRLLTALHLQHINAARHLQKPTCFQN
QTGWTGNWLVDWSGTRLKTG

>GaADK7

MASSSSVNLEDVPSESLMTELLRRMKCATKPEKRLILIGPPGSGKGTQSPMIKDEYCLCHLATGDILRA
AVAAKTPLGVKAKEAMDKGELVSDELVVGIIDEAMKKPSCQKGFILDGFPRTVGQAQMLDEMLEKQ
GVKIDKVLDFAIDDSILEERITGRWIHPASGRSYHTKFAPPKVPGVDDVTGEPLIQRKDDTAAVLKSRLD

AFHKQTEPVIDYYWKKGIVAKLHAAKSPKEVTEEVQKVLS
>GaADK8
MAIACLPKLRLATAPAVSVSTFLKPLRHGLRSFGSAAALELPDEDEYYEYEQAHRDRRSAQPKIDVDGS
ATDRGVQWVLIGEPGVKRHAYAERLSKLLEVPHISMGTLVRQELNPHSSLYKQIANAVNEGKLVPEDV
IFALLSKRLEEGYYGGENGFILDGIPRTRIQAEILDQITDIDLVVNFKCTEEYMLKSSESEVLHIGSSKDVGT
SWKKNVHVYSEQAKSVEDYYSKQKKLLNFQVSAAPADAWQGLLAALHLQHINALTSSQKLTA
>GaADK9
MASSSVNLEEIPSESLMNELLRRMKCAPKPDKRLILIGSLFFPFIFIFMSLCLCPLALGDMLRALVSTKTPL
GIKAKKAMDKKPSCKKGFILDGFPRTVAQAQKLSRLLRILQVVSLFDCDVKIEDYLLKSYYKTASLIAAST
KGAAIFDWSDRSNLGLSFQVVDDILDFTQSAEQLGKPAGSDLTKGNLTAQVINMA
>GaADK10
MATNSMNLEDVPSESLMSELLRRMKCASKPDKRLILIGPPGSGKGTQSPIIKDEYCLCHLATGDMLRA
AVAAKTPLGIKAKEAMDKGELVSDDLVVGIIDEAMKKPSCKKGFILDGFPRTVGQAQKLDDMLEKQGI
KIDKVLDFAIDDAVLEERITGRWIHPASGRTYHTKFAPPKVPGLDDVTGEPLVQRKDDTAAVLKSRLDA
FHKQTQPVIDYYSEKGIVAKLHAEKPQKEVTDEVKKVLS
>GaADK11
MAVLSRCSRAVTAATTPNAAAAKFSYLIRALSPFSSSSSSSSSSTFDADNGLNSRIKPAVLGTLKREPKD
RNVQWVFLGCPGVGKGTYASRLSDLLGIPHIATGDLVRDELASSGPLSSQLKEIVNQGKLVSDEIIIDLL
SKRLEAGEAKGESGFILDGFPRTVRQAEILEGVTDIDLVINLKLREEALLAKCLGRRICSECGGNYNVACI
DIKAENGKPGMYMAPLPPPPQCASKLITRADDTEEVVKQRLRIYQAMTLPVEDYYRSLGKLLEFDLPG
GVRESWPKLLHALNLEDEEDKQSAAA
>GaADK12
MAVVSRCSRAATAATTANATTATTFAYLIRSLSPFSSSSSTFDPQNDLNNRNKPAVLGTLKRESKGRN
VQWVFLGCPGVGKGTYASRLSNLLGVPHIATGDLVRDELASSGPLSSQLKEIVTQGKLVSDEIIIDLLSK
RLEAGEAKGESGFILDGFPRTVRQAEILEGVTDIDLVINLKLREEALLAKCLGRRICSECGGNYNVACIDI
KAENGRPGMYMAPLPPPPQCASKLITRPDDTEEVVKQRLRIYQAMTRPVEDFYRSRGKLLEFDLPGGI
PESWPKLLCALNLEDREDKQSAAA
>GaADK13
MNELLRRMKCAPKPEKHLILIGPPGSGKGTQSPIIKDEHCLCPLATGDMLRAVVSSKTALCIKAKEAMD
KCLAIVSQGELVFDDLVVGIIDEAIKKPSCKKGFILDGFPRTVAQAQKLSRLLTILQVVSLFDFDVKLKDYL
LKSYYKTTSLIAASTKGAVIFSGADHSVTEQMYEYGKNLGLSFQVVDDILDFTQSAEQLGKPAGSDLAK
GNLTVQVINMA
>GaADK14
MWRRVASLSSSISSSFLAQTASRLNTWESLTTGIAQQAKGVIAPKEKTPFITFVLGGPGSGKGTQCIKIV
ETFGFAHLSAGDLLRREIASKSADGAMILNTIKEGKIVPSQVTVKLIQKEMELSDNHKFLIDGFPRTEENR
VSFERIIGVEPNVVLFFDCPEEEMVKRVLNRNEGRVDDNLETIKKRLTVFQALSLPVINYYSERGKLYTIK
AIGTEDEIFEQVRPIFAAFETVG
>GaADK15
MASSSVNLEEIPSESLMNELLRRMKCAPKPEKHLILIGPPGSGKGTQSPIIKDEHCLCHLATGDMLRAA
VSAKTPLGIKAKESMDKGELVSDDLVVGIIDEAMKKPSCKKGFILDGFPRTVAQAQKLDEMLERQGVKI
DKVLDFAIDDAVLEERISGRWIHPASGRSYHTKFAPPRVPGVDDVTGGPLIQRKDDTAAVLKSRLESFH
RQTQPVINYYSKKGIVATLHAEKPLSDVRDEVRKVLS
>GaADK16
MGTVSVANKDINVSLAEKKPRVVFVLGGPGSGKGTQCANIVEHFHYTHLSAGDLLRAEIKSGSENGT

MIQNMIKEGKIVPSEVTIKLLQKAMLESDNDKFLIDGFPRNEENRAAFEAVTKIEPEFVLFFNCPEEEME
KRLLSRNQGREDDNIETIRKRFNVFLESSLPVIEHYKAKEKVREIEAAKPIGEVFEAVKVVFTPKAEKVVA

G. barbadense

>GbADK1
MADSAVALEDVASVDIMTELLRRFKCSSKPDKRLILIGPPGSGKGTQSPMIKDDYCLCHLATGDMLRA
AVAAKTPLGIKAKEAMDKGELVSDDLVVGIIDEAMKKTSCQKGFILDGFPRTVVQAQKLDEMLEKQG
TKIDKVLNFAIDDSILEERITGRWIHPSSGRTYHTKFAPPKVPGLDDVTGEPLIQRRDDTPAVLKSRLEAF
HRQTEPVIHYYGSKGILANLPAEKPPKEVTSEVQKVLSS

>GbADK2
MGSTSFSPSISLSPSLSPHSSKFPNPSPNLFLRSHSSFTPLTLRSNQTRYRFKKLPSSNAPNFLVAASAKA
EPLKVIISGAPASGKGTQCELITQKYGLVHIAAGDLLRAEVAAASENGKLAKEYMEKGELVPDEIVVMM
VKERLLQPDSQQRGWLLDGYPRSSSQAAALEDYGIRPDVFILLDVSEDILVERVVGRRLDPLTGKIYHL
KYSPPENNEIASRLTQRFDDTEEKACLIFLVKLRLRTHHQNVDAVLSVYKDITVNGNEAKERVFAQIDA
ALTHVGEQRKMETRVEMKKKKKKKKKKKKKRSCMTSMI

>GbADK3
MAWLPKLRVASASVASSAVNPLRRWIRDIGSAAALELDYDYDYYEYERAPRDRRLPQQKLDVDGSVP
ERGVQWVLIGEPGVKRHAYAERLSKLLGIPHISMGSLIRQELDPYSSLYKQVVNAVNEGKLVPEDVVFA
LLSKRLEEGENGFILDGIPRTRLQAEILDQIVDIDLVVNFKCSEEYMKMSSESELLHFGNSKGVESSLKKL
RVYSEQAKSVEDYYSKQKKLLNFQLAGATGDAWQGLLAALHLQYSPINALSSSPKLTA

>GbADK4
MGTANDSSKDISDRLAEKHPKVVFVLGGPGSGKGTQCEKIVQHFGYTHLSAGDLLRAEKNSGSENGI
MIQNMMNEGKIVPSEVTIKLLQKAMLDSGNEKFLIDGFPRNEENRAAFEALTKIEPEFVLYFNCPEKEM
ERRILNRNQGREDDNIETIRKRFKVFLESSLPVIEYYKGKGKVREIDAAKPVEEVFEEVKDIFNPKVPKDE
NVEGRCMCCAIL

>GbADK5
MLASSSSTAISLHPLANYNASATPSLNPSSLSFSSSYSYLSPLSFRLHSSKTHLRITPKKGLRVSCSTNEPL
KVMISGAPASGKGTQCELIVQKFGLVHISTGDLLRAEVSSGTEIGNKAKEFMNSGRLVPDEIVTAMVTV
RLSRQDAKEKGWLLDGYPRSFAQAQSLEELNIRPDIYIVLDVPDEILIDRCVGRRLDPVTGKIYHLKNFP
PESEEIKARLVTRADDTEEKVKSRLEIYKQNAAAISSTYSSITNKIDGNRPKEIIFKDIDSLLSQLLKDKTAK
SMKPVLQTENCLGQASSNEEKWRGIPTRLNNIPHSREIRNYFYDDVLQATQRAVNDGRTRLKVEINIP
ELNPEMDVYRIGTLMELVRVIALSFADDGKRVKVCVQGSMGEGALAGMPLQLAGTRKILEFMDWGN
YGAMGTFIKIGSIGAKEVDEEDDMFILVAPQNAVGNCIIDDLRAMTDAAGKRPVILINPRLKDLPGSSG
IMQTMGRDKRLEYAASFESCYFFRLLYYAGTQYPIMGAIRMTYPYDYELYKRVDEPSGKEKYVSLSTFKE
RPTIDEINDAFLGKPRNKDKKASGIWGFLSGVF

>GbADK6
MIINKLSHLATVTTATTAAVTAAPPLYRLLKLTDSLFHGAAQPQPDTDYWYYYPPSQQESRRDELVRS
TPVADTDGSVPLRGVQWAFIGSPRAKKRVYAVMLSKLLEVPHITMASLVRQELSPNSNLYKQIANAV
DHRELVNEDIILGLLSKRLEDGHYRGETGFILDGIPRSRTQAEILDQLAEIDLVVNFKCTEDLMMNNQG
EASWTERLQDYIKQSKVVEEYYKNQKKLLEFQIGNASMETWRRLLTALHLQHINAARHLQKPTCFQN
QTGWTRNWLVDWSGTRLKTG

>GbADK7
MASSSSVNLEDVPSESLMTELLRRMKCATKPEKRLILIGPPGSGKGTQSPMIKDEYCLCHLATGDMLR
AAVAAKTPLGVKAKEAMDKGELVSDELVVGIIDEAMKKPSCQKGFILDGFPRTVGQAQMLDEMLEK
QGVKIDKVLDFAIDDSILEERITGRWIHPASGRSYHTKFAPPKVPGVDDVTGEPLIQRKDDTAAVLKSRL

DAFHKQTEPVIDYYWKKGIVAKLHAAKSPKEVTEEVQKVLS
>GbADK8
MAIACLPKLRLATAPAVSVSTFLKPLRHGLRSFGSAAALELPDEDEYYEYEQAHRDRRSAQPKIDVDGS
ATDRGVQWVLIGEPGVKRHAYAERLSKLLEVPHISMGTIVRQELNPHSSLYKQIANAVNEGKLVPEDV
IFALLSKRLEEGYYGGENGFILDGIPRTRIQAEILDQITDIDLVVNFKCTEEYMLKSSESEVLHIGSSKDVGT
TWKKNVHVYSEQAKSVEDYYSKQKKLLNFQVSAAPADAWQGLLAALHLQHINALTSSQKLTA
>GbADK9
MASSSVNLEEIPSESLMNELLRRMKCAPKPDKRLILIGPPGSGKGTQSPIIKDEHCLCPLATGDMLRAA
VSTKTPLGIKAKKAMDKGDLISDDLVVGIIDEAMKKPSCKKGFILDGFPRTVAQSQKLSRLLRILQVVSLF
DCDVKVEDYLLKSYYKTASLIAASTKGAAIFSGADRSVTEQMYEYGKNLGLSFQVVDDILDFTQSAEQL
GKPAGSDLTKGNLTAQVINMA
>GbADK10
MATNSMNLEDVPSESLMSELLRRMKCASKPDKRLILIGPPGSGKGTQSPIIKDEYCLCHLATGDMLRA
AVAAKTPLGIKAKEAMDKGELVSDELVVGIIDEAMKKPSCKKGFILDGFPRTVGQAQKLDDMLEKQGV
KIDKVLDFAIDDAVLEERITGRWIHPASGRTYHTKFAPPKVPGLDDVTGEPLVQRKDDTAAVLKSRLDA
FHKQTQPVIDYYSEKGIVAKLHAEKPQKEVTDEVKKVLS
>GbADK11
MAVLSRCSRAVTAATTPNAAAAKFSYLIRALSPFSSSSSSSSTFDADNGLNSRIKPAVLGTLKREPKDR
NVQWVFLGCPGVGKGTYASRLSDLLGIPHIATGDLVRDELASSGPLSSQLKEIVNQGKLVSDEIIIDLLS
KRLEAGEAKGESGFILDGFPRTVRQAEILEGVTDIDLVINLKLREEALLAKCLGRRICSECGGNYNVACID
IKAENGKPGMYMAPLPPPPQCASKLITRADDTEEVVKQRLRIYQAMTLPVEDYYRSRGKLLEFDLPGG
VRESWPKLLHALNLEDEEHKQSAAA
>GbADK12
MAVVSRCSRAATAATTANATTATTFTYLIRSLSPFSSSSSTFDPQNDLNNRNKPAVLGTLKRESKGRN
VQWVFLGCPGVGKGTYASRLSNLLGVPHIATGDLVRDELASSGPLSSQLKEIVTQGKLVSDEIIIDLLSK
RLEAGEAKGESGFILDGFPRTVRQAEILEGVTDIDLVINLKLREEALLAKCLGRRICSECGGNYNVACINI
KAENGRPGMYMAPLPPPPQCASKLITRPDDTEEVVKQRLRIYQAMTRPVEDFYRSRGKLLEFDLPGGI
PESWPKLLCALNLEDREDKQSAAA
>GbADK13
MVLDSARLKMSAPCRVVEFHAAMIKQSLNLKYKYCLHSMEFGLLESPPGSGKGTQSPIIKDEHCLCPL
ATGDMLRAVVSSKTALSIVSQGELVYDDLVVGIIDEAIKKPSCKKGFILDGFPRTVAQAQKVIL
>GbADK14
MTSSSVNLEEIPSESLMNELLRRMKCAPKPDKRLILIGPPGSGKGTQSPIIKYEHCLCSLATGDMLRAAV
SAKTPLGIKAKKAMDKGELISDDLVVGIIDEAMNKPSHKKGFILDGFPRTVAQAQKDHLHHKIAQCYIS
TGEKVLAIQFIRKTSTKGAGIFSGANRSVTEQMYEYGKNLGLLFQVVDDILDFTQSAEQLGKPAASASP
STSTRRKPKSAWNLIAWTQARASAANVGPPDTQFGPYCNGKRAVFKSMICGLTAINASRAVGCISKS
SLV
>GbADK15
MACSSVNLEEIPSESLMNELLHRMKCAPKPDKCFILIGPPGFGKGTQSPIIKDEHCLWLSQCPLATGDM
LRAAVFAKTPLGIKAKKAMDKGELISDDLVVGIIDEAMKKPLCKKGFIFDGFPRTVAQAQKVIL
>GbADK16
MASSSVNLEEIPSESLMNELLRRMKCAPKPEKHLILIGPPGSGKGTQSPIIKDEHCLCHLATGDMLRAA
VSAKTPLGIKAKESMDKGELVSDDLVVGIIDEAMKKPSCKKGFILDGFPRTVAQAQKLDEMLERQGVKI
DKVLDFAIDDAVLEERISGRWIHPASGRSYHTKFAPPRVPGVDDVTGEPLIQRKDDTAAVLKSRLESFH

RQTLPVINYYSKKGIVATLHAEKPLSDVTDEVRKVLS
>GbADK17
MWRRVASLSSSISSSFLAQTASRLNTWESLTTGIAQQAKGVIAPKEKTPFITFVLGGPGSGKGTQCIKIV
ETFGFAHLSAGDLLRREIASKSADGAMILNTIKEGKIVPSQVTVKLIQKEMELSDNHKFLIDGFPRTEENR
VSFERIIGVEPNVVLFFDCPEEEMVKRVLNRNEGRVDDNLETIKKRLTVFQALSLPVINYYSERGKLYTIK
AIGTEDEIFEQVRPIFAAFETVG
>GbADK18
MGTVSVANKDINVSLAEKKPRVVFVLGGPGSGKGTQCANIVEHFHYTHLSAGDLLRAEIKSGSENGT
MIQNMIKEGKIVPSEVTIKLLQKAMLESDNDKFLIDGFPRNEENRAAFEAVTKIEPEFVLFFNCPEEEME
KRLLSRNQGREDDNIETIRKRFNVFLKSSLPVIEHYKAKEKVREIEAAKPIGEVFEAVKVVFTPKAEKVVA
>GbADK19
MADSAVALEDVASVDIMTELLRRFKCSSKPDKRLILIGPPGSGKGTQSPMIKDDYCLCHLATGDMLRA
AVAAKTPLGIKAKEAMDKGELVSDDLVVGIIDEAMKKPSCQKGFILDGFPRTVVQAQKLDEMLGKQG
TKIDKVLNFAIDDSILEERITGRWIHPSSGRTYHTKFAPPKVPGLDDVTGEPLIQRKDDTPAVLKSRLEAF
HRQTEPVIDYYGSKGILTNLPAEKPPKEVTSEVQKVLSS
>GbADK20
MGTANDSSKDISVRLAEKKPKVVFVLGGPGSGKGTQCEKIVQDFGYTHLSAGDLLRAEKNSGSENGI
MIQNMMNEGKIVPSEVTIKLLQKAMLESGNDKFLIDGFPRNEENRAAFEAVTKIEPEFVLYFNCSEKEM
ERRILNRNQGREDDNIETIRKRFKVFLESSLPVIEYYKGKGKVREIDAAKPVEEVFEEVKGIFNPPEVRKVV
GGRGWGRGCCAIL
>GbADK21
MAWLPKLRVATASAASSAVNPLRRWIRDIGSAAALELDYDYDYYDYERAALDRRVPQQKLDVDGSV
PERGVQWVLIGEPGVKRHSYAERLSKLLGIPHISMGSLIRQELDPYSSVYKQVVNAVNEGKLVPEDVVF
ALLSKRLEEGENGFILDGIPRTRLQAEILNQIVDIDLVVNFKCSEEYMLKMSSESEILHIGNSKGVESSLKK
KLRIYSEQAKSVEDYYSKQKKLLNFQLAGATGDAWQGLLAALHLQYSPINALSSSPKLTA
>GbADK22
MLIMGSTSFSPSLSLSPSLSPHSSKFPNPNPNLFLRSHSSFTPLTLRSNQTRYRFKKLPSSNAPNFLVAAS
GKAEPLKVIISGAPASGKGTQCELITQKYGLVHIAAGDLLRAEVAAASENGKLAKEYMEKGELVPNEIV
VMMVKERLLQPDSQQRGWLLDGYPRSSSQAAALEDYGIRPDLFILLDVSEDILVERVVGRRLDPLTGK
IYHLKYSPPENDEIASRLTQRFDDTEEKVKLRLRTHHQNVDAVLSVYKDITVKVNGNEAKEHVFAQINA
ALTHVGGGESFKADLTILTCNQNLIERTCKQTPFYDLCIWSLKSNPESRDASVKKLAQIMVDSLKTKAT
ETLDLIDELLQDGLALDPEMQKALTSCAERYNVIIRGDVPEINEALKTGDYKFAAKGANDAAIEANSCEI
EFPTKSPLTDMNKVVHDVSVVAASIVKIIQTKFSF
>GbADK23
MLATSSSSSSSTAISLHPLTNYNASATPSLNPSSLSFSSSYSYLSPLSFRLHSSKTHLRITPKKGLRVSCST
NEPLKVMISGAPASGKGTQCELIVQKFGLVHISTGDLLRAEVSSGTEIGNKAKEFMNSGRLVPDEIVTA
MVTVRLSRQDAKEKGWLLDGYPRSFAQAQSLEELNIRPDIYIVLDVPDEILIDRCVGRRLDPVTGKIYHL
KNFPPESEEIKARLVTRADDTEEKVKSRLEIYKQNAAAISSTYSSITNKIDGNRPKEMIFKDIDSLLSQLLK
DKTVKSVKPVLQTESRLDQASSNQEKWRGIPTRLNNIPHSREIRNYFYDDVLQATQRAVNDGRTRLK
VEINIPELNPEMDVYRIGTLMELVRVIALSFADDGKRVKVCVQGSMGEGALAGMPLQLAGTRKILEFM
DWGNYGAMGTFIKIGSIGAKEVDEEDDMFILVAPQNAVGNCIIDDLRGMTDAAGKRPVILINPRLKDL
PGSSGIMQTMGRDKRLEYAASFESCYFFRLLYYAGTQYPIMGAIRMTYPYDYELYKRVDEPSGKEKYVS
LSTFKERPTIDEINDVFLGKPRNKDKKASGIWGFLSGVF
>GbADK24

MIINKLSHLATVTAAAVTAGPPLYRLLKLTVSRFHGAAQPQPDTDYWYYYHVSPSQQESCRDELFRST
PVADTNGSVPLRGVQWAFIGSPRAKKRVYAVMLSKLLEVPHITMASLVRQELSPNSNLYKQIANAVD
HGEPVNEDIILGLLSKRLEDGHYRGETGFILDGIPRSRIQAEILDQLAEIDLVVNFKCTEDLMMNNQGEA
SWTERLQDYIKQSKVVEDYYKNEKKLLEFQIGNARMETWRRLLTALHLQHINAARLLQKPTNQTGWT
GNWLVDWSRKRLKAG
>GbADK25
MASSSSVNLEDVPSESLMTELLRRMKCATKPEKRLILIGPPGSGKGTQSPMIKDEYCLCHLATGDMLR
AAVAAKTPLGVKAKEAMDKGELVSDELVVGIIDEAMKKPSCQKGFILDGFPRTVGQAQMLDEMLEK
QGVKIDKVLDFAIDDSILEERITGRWIHPASGRSYHTKFAPPKVPGVDDVTGEPLIQRKDDTAAVLKSRL
EAFHKQTEPVIDYYWKKGVVAKLHAEKSPKEVTEEVQKENLGKRIFNNGKTIRKDNQEPIHEFNTESKIT
NSVLLRFTTINIPFFFQENIPIPLHFHPFFLIFLHFLGNLHFQILP
>GbADK26
MARACLPKLRLATATAVSVSTFLKPLRHGLRSFGSAAALELPDEDVYYEYEQAHRDRRSAQPKIDVDG
SATDRGVQWVLIGEPGVKRHAYAERLSKLLEVPHISMGTLVRQELNPHSSLYKQIANAVNEGKLVPED
VIFALLSKRLEEGYYGGENGFILDGIPRTRIQAEILDQITDIDLVVNFKCTEEQMLKSSESEVLHIGSSKNV
GTSWKKNVHVYSEQAKSVEDYYSKQKKLLNFQVSGAPADAWQGLLAALHLQHINALTSSQKLTA
>GbADK27
MATNSMNLEDIPSESLMSELLRRMKCASKPDKRLILIGPPGSGKGTQSPIIKDEYCLCHLATGDMLRAA
VAAKTPLGIKAKEAMDKGELVSDDLVVGIIDEAMKKPSCKKGFILDGFPRTVGQAQKLDDMLGKQGV
KIDKVLDFAIDDAVLEERITGRWIHPASGRTYHTKFAPPKVPGLDDVTGEPLVQRKDDTAAVLKSRLDA
FHKQTQPVIDYYSEKGIVAKLHAEKPQKEVTDEVKKVLS
>GbADK28
MAVLSRCSRAVTAATTPNAAAAKFSYLIRALSPFSSSSSTFDADNGLNSRIKPAVLGTLKREPKDRNVQ
WVFLGCPGVGKGTYASRLSDLLGIPHIATGDLVRDELASSGPLSSQLKEIVNQGKLVSDEIIIDLLSKRLE
AGEAKGESGFILDGFPRTVRQAEILEGVTDIDLVINLKLREEALLAKCLGRRICSECGGNYNVACIDIKAE
NGKPGMYMAPLPPPPQCASKLITRADDTEEVVKQRLRIYQAMTLPVEDFYRCRGKLLEFDLPGGVRES
WPKLLHALNLEDEEDKQSAAA
>GbADK29
MAVVSRCSRAATAATTANATTAPTFAYLIRSLSPFSSSSSTFDPQNDLNNRNKPAVLGTLKRESKGRN
VQWVFLGCPGVGKGTYASRLSNLLGVPHISTGDLVRDELASSGPLSSQLKEIVTQGKLVSDEIIIDLLSK
RLEAGEAKGESGFILDGFPRTVRQAEILEGVTDIDLVINLKLREEALLAKCLGRRICSECGGNYNVACIDI
KAENGRPGMYMAPLPPPPQCASKLITRPDDTEEVVKQRLRIYQAMTRPVEDFYRSRGKLLEFDLPGGI
PESWPKLLCALNLEDREDKQSAAA
>GbADK30
MASGSVNLEEIPYESLMNELLRRMKCAPKPEKRLILIGPPGSGKGTQSPIIKDEHCLCHLAAGDMLRAA
VSAKTPLGIKAKEAMDKGELVSDDLVVGIIDEAMKKPSCKKGFILDGFPRTVAQAQKLDEMLERQGVK
IDKVLDFAIDDAVLEERISGRWIHPASGRSYHTKFAPPRVPGVDDVTGEPLIQRKDDSAAVLKSRLESFH
RQTQPVINYYSKKGIVATLHAEKPLTDVTDEVRKVLS
>GbADK31
MWRRVASLSSSISSSFLAQTASRLNTWESLTTGIAQQAKGVIAPKEKTPFITFVLGGPGSGKGTQCIKIV
ETFGFAHLSAGDLLRREIASKSADGAMILNTIKEGKIVPSQVTVKLIQKEMELSDNHKFLIDGFPRTEENR
VSFEQIIGVEPNVVLFFDCPEEEMVKRVLNRNEGRVDDNLETIKKRLTVFQALSLPVINYYSEKGKLYTIK
AIGTEDEIFEQVRPIFAAFETVG
>GbADK32

MGAVNVANKDINVSLAEKKPRVVFVLGGPGSGKGTQCANIVEHFHYTHLSAGDLLRAEIKSGSENGT
MIQNMIKEGKIVPSEVTIKLLEKAMLESGNDKFLIDGFPRNEENRAAFEAVTKIEPEFVLFFNCPEEEMEK
RLLSRNQGREDDNIETIRKRFKVFLDSSLPVIEYYKVKGKVREIEAAKPIGEVFEAVKVVFTPKAEKVVA
G. hirsutum
>GhADK1
MGSTSFSPSISLSPPLSPHSSKFPNPSPNLFLHSHSSFTPLTLRSNQTRYRFKKLPSSNAPNFLVAASAKA
EPLKVIISGAPASGKGTQCELITQKYGLVHIAAGDLLRAEVAAASENGKLAKEYMEKGELVPDEIVVMM
VKERLLQPDSQQRGWLLDGYPRSSSQAAALEDYGIRPDVFILLDVSEDILVERVVGRRLDPLTGKIYHL
KYSPPENDEIASRLTQRFDDTEEKACLIFLVKLRLRTHHQNVDAVLSVYKDITVNGNEAKEHVFAQIDA
ALTHVGEQRKVNSGSVAA
>GhADK2
MAWLPKLRVASASVASSAVNPLRRWIRDIGSAAALELDYDYDYYEYERAPRDRRLPQQKLDVDGSVP
ERGIQWVLIGEPGVKRHAYAERLSKLLGIPHISMGSLIRQELDPYSSLYKQVVNAVNEGKLVPEDVVFA
LLSKRLEEGENGFILDGIPRTRLQAEILDQIVDIDLVVNFKCSEEYMKMSSESELLHIGNSKGVESSLKKLR
VYSEQAKSVEDYYSKQKKLLNFQLAGATGDAWQGLLAALHLQYSPINALSSSPKLTA
>GhADK3
MGTANDSSKDISDRLAEKHPKVVFVLGGPGSGKGTQCEKIVQHFGYTHLSAGDLLRAEKNSGSENGI
MIQNMMNEGKIVPSEVTIKLLQKAMLDSGNDKFLIDGFPRNEENRAAFEALTKIEPEFVLYFNCPEKEM
ERRILNRNQGREDDNIETIRKRFKVFLESSLPVIEYYKGKGKVREIDAAKPVEEVFKEVKDIFNPKVPKVPK
DENVEGRCMCCAIL
>GhADK4
MLASSSSTAISLHPLANYNASATPSLNPSSLSFSSSYSYLSPLSFRLHSSKTHLRITPKKGLRVSCSTNEPL
KVMISGAPASGKGTQCELIVQKFGLVHISTGDLLRAEISSGTEIGNKAKEFMNSGRLVPDEIVTAMVTV
RLSRQDAKEKGWLLDGYPRSFAQAQSLEELNIRPDIYIVLDVPDEILIDRCVGRRLDPVTGKIYHLKNFP
PESEEIKARLVTRADDTEEKVKSRLEIYKQNAAAISSTYSSITNKIDGNRPKEIIFKDIDSLLSQLLKDKTAK
SVKPVLQTENCLDQASSNEEKWRGIPTRLNNIPHSREIRNYFYDDVLQATQRAVNDGRTRLKVEINIP
ELNPEMDVYRIGTLMELVRVIALSFADDGKRVKVCVQGSMGEGALAGMPLQLAGTRKILEFMDWGN
YGAMGTFIKIGSIGAKEVDEEDDMFILVAPQNAVGNCIIDDLRAMTDAAGKRPVILINPRLKDLPGSSG
IMQTMGRDKRLEYAASFESCYFFRLLYYAGTQYPIMGAIRMTYPYDYELYKRVDEPSGKEKYVSLSTFKE
RPTIDEINDAFLGKPRNKDKKASGIWGFLSGVF
>GhADK5
MIINKLSHLATVTTATTAAVTAAPPLYRLLKLTDSLFHGAAQPQPDTDYWYYYPPSQQESRRDELVRS
TPVADTDGSVPLRGVQWAFIGSPRAKKRVYAVMLSKLLEVPHITMASLVRQELSPNSNLYKQIANAV
VHGELVNEDIILGLLSKRLEDGHYRGETGFILDGIPRSRTQAEILDQLAEIDLVVNFKCTEDLMMNNQG
EASWTERLQDYIKQSKVVEEYYKNQKKLLEFQIGNASMETWRRLLTALHLRHINAARHLQKPTVNESI
V
>GhADK6
MASSSSVNLEDVPSESLMTELLRRMKCATKPEKRLILIGPPGSGKGTQSPMIKDEYCLCHLATGDMLR
AAVAAKTPLGVKAKEAMDKGELVSDELVVGIIDEAMKKPSCQKGFILDGFPRTVGQAQMLDEMLEK
QGVKIDKVLDFAIDDSILEERITGRWIHPASGRSYHTKFAPPKVPGVDDVTGEPLIQRKDDTAAVLKSRL
DAFHKQTEPVIDYYWKKGIVAKLHAAKSPKEVTEEVQKVLS
>GhADK7
MAIACLPKLRLATAPAVSVSTFLKPLRHGLRSFGSAAALELPDEDEYYEYEQAHRDRRSAQPKIDVDGS
ATDRGVQWVLIGEPGVKRHAYAERLSKLLEVPHISMGTLVRQELNPHSSLYKQIANAVNEGKLVPEDV

IFALLSKRLEEGYYGGENGFILDGIPRTRIQAEILDQITDIDLVVNFKCTEEYMLKSSESEVLHIGSSKDVGT
TWKKNVHVYSEQAKSVEDYYSKQKKLLNFQVSAAPADAWQGLLAALHLQHINALTSSQKLTA
>GhADK8
MASSSVNLEEIPSESLMNELLRRMKCAPKPDKRLILIGPPGSGKGTQSPIIKDEHCLCPLATGDMLRAA
VSTKTPLGIKAKKAMDKGDLISDDLVVGIIDEAMKKPSCKKGFILDGFPRTVAQSQKLSRLLRILQVVSLF
DCDVKVEDYLLKSYYKTASLIAASTKGAAIFSGADRSVTEQMYEYGKNLGLSFQVVDDILDFTQSAEQL
GKPAGSDLTKGNLTAQVINMA
>GhADK9
MATNSMNLEDVPSESLMSELLRRMKCAYKPDKRLILIGPPGSGKGTQSPIIKDEYCLCHLATGDMLRA
AVAAKTPLGIKAKEAMDKGELVSDDLVVGIIDEAMKKPSCKKGFILDGFPRTVGQAQKLDDMLEKQG
VKIDKVLDFAIDDAVLEERITGRWIHPASGRTYHTKFAPPKVPGLDDVTGEPLVQRKDDTAAVLKSRLD
AFHKQTQPVIDYYSEKGIVAKLHAEKPQKEVTDEVKKVLS
>GhADK10
MAVLSRCSRAVTAATTPNAAAAKFSYLIRALSPFSSSSSSSSTFDADNGLNSRIKPAVLGTLNREPKDR
NVQWVFLGCPGVGKGTYASRLSDLLGIPHIATGDLVRDELASSGPLSSQLKEIVNQGKLVSDEIIIDLLS
KRLEAGEAKGESGFILDGFPRTVRQAEILEGVTDIDLVINLKLREEALLAKCLGRRICSECGGNYNVACID
IKAENGKPGMYMAPLPPPPQCASKLITRADDTEEVVKQRLRIYQAMTLPVEDYYRSRGKLLEFDLPGG
VRESWPKLLHALNLEDEEHKQSAAA
>GhADK11
MASSSVNLEEIPSESLMNELLRRMKCAPKPEKHLILIGPPGSGKGTQSPIIKDEHCLCHLATGDMLRAA
VSAKTPLGIKAKESMDKGELVSDDLVVGIIDEAMKKPSCKKGFILDGFPRTVAQAQKLDEMLERQGVKI
DKVLDFAIDDAVLEERISGRWIHPASGRSYHTKFAPPRVPGVDDVTGESLIQRKDDTAAVLKSRLESFH
RQTQPVINYYSKKGIVATLHAEKPLSDVTDEVRKVLS
>GhADK12
MWRRVASLSSSISSSFLAQTASRLNTWESLTTGIAQQAKGVIAPKEKTPFITFVLGGPGSGKGTQCIKIV
ETFGFAHLSAGDLLRREIASKSADGAMILNTIKEGKIVPSQVTVKLIRKEMELSDNHKFLIDGFPRTEENR
VSFERIIGVEPNVVLFFDCPEEEMVKRVLNRNEGRVDDNLETIKKRLTVFQALSLPVINYYSERGKLYTIK
AIGTEDEIFEQVRPIFAAFEQTVG
>GhADK13
MGTVSVANKDINVSLAEKKPRVVFVLGGPGSGKGTQCANIVEHFHYTHLSAGDLLRAEIKSGSENGT
MIQNMIKEGKIVPSEVTIKLLQKAMLESDNDKFLIDGFPRNEENRAAFEAVTKIEPEFVLFFNCPEEEME
KRLLSRNQGREDDNIETIRKRFNVFLESSLPVIEHYKAKEKVREIEAAKPIGEVFEAVKVVFTPKAEKVKKS
IGFSLLTLENNRTSSIHGRYALHIQVVA
>GhADK14
MADSAVALEDVASVDIMTELLRRFKCSSKPDKRLILIGPPGSGKGTQSPMIKDDYCLCHLATGDMLRA
AVAAKTPLGIKAKEAMDKGELVSDDLVVGIIDEAMKKPSCQKGFILDGFPRTVVQAQKLDEMLGKQG
TKIDKVLNFAIDDSILEERITGRWIHPSSGRTYHTKFAPPKVPGLDDVTGEPLIQRKDDTPAVLKSRLEAF
HRQTEPVIDYYGSKGILANLPAEKPPKEVTSEVQKVLSS
>GhADK15
MQDISVRLAEKKPKVVFVLGGPGSGKGTQCEKIVQDFGYTHLSAGDLLRAEKNSGSENGIMIQNMM
NEGKIVPSEVTIKLLQKAMLESGNDKFLIDGFPRNEENRAAFEAVTKIEPEFVLYFNCSEKEMERRILNRN
QGREDDNIETIRKRFKVFLESSLPVIEYYKGKGKVREIDAAKPVEEVFEEVKGIFNPPEVRKVVGGRGWG
RGCCAIL
>GhADK16

MAWLPKLRVATASAASSAVNPLRRWIRDIGSAAALELDYDYDYYDYERAALDRRVPQQKLDVDGSV
PERGVQWVLIGEPGVKRHSYAERLSKLLGIPHISMGSLIRQELDPYSSLYKQVVNAVNEGKLVPEDVVF
ALLSKRLEEGENGFILDGIPRTRLQAEILDQIVDIDLVVNFKCSEEYMLKMSSESEILHIGNSKGVESSLKK
KLRVYSEQAKSVEDYYSKQKKLLNFQLAGATGDAWQGLLAALHLQYSPINALSSSPKLTA
>GhADK17
MLIMGSTSFSPSLSLSPSLSPHSSKFPNPNPNLFFRSHSSFTPLTLRSNQTRYRLKKLPSSNAPNFLVAAS
GKAEPLKVIISGAPASGKGTQCELITQKYGLVHIAAGDLLRAEVAAASENGKLAKEYMEKGELVPNEIV
VMMVKERLLQPDSQQRGWLLDGYPRSSSQAAALEDYGIRPDLFILLDVSEDILVERVVGRRLDPLTGK
IYHLKYSPPENDEIASRLTQRFDDTEEKACLIFLVKLRLRTHHQNVDAVLSVYKDITVNGNEAKEHVFA
QIDAALTHVGEQRKVNSGSLAA
>GhADK18
MLATSSSSSSSTAISLHPLTNYNASATPSLNPSSLSFSSSYSYLSPLSFRLHSSKTHLRITPKKGLRVSCST
NEPLKVMISGAPASGKGTQCELIVQKFGLVHISTGDLLRAEVSSRTEIGNKAKEFMNSGRLVPDEIVTA
MVTVRLSRQDAKEKGWLLDGYPRSFAQAQSLEELNIRPDIYIVLDVPDEILIDRCVGRRLDPVTGKIYHL
KNFPPESEEIKARLVTRADDTEEKVKSRLEIYKQNAAAISSTYSSITNKIDGNRPKEMIFKDIDSLLSQLLK
DKTVKSVKPVLQTESRLDQASSNQEKWRGIPTRLNNIPHSREIRNYFYYDVLQATQRAVNDGRTRLK
VEINIPELNPEMDVYRIGTLMELVRVIALSFADDGKRVKVCVQGSMGEGALAGMPLQLAGTRKILEFM
DWGNYGAMGTFIKIGSIGAKEVDEEDDMFILVAPQNAVGNCIIDDLRGMTDAAGKRPVILINPRLKDL
PGSSGIMQTMGRDKRLEYAASFESCYFFRLLYYAGTQYPIMGAIRMTYPYDYELYKRVDEPSGKEKYFS
LSTFKERPTIDEINDAFLGKPRNKDKKASGIWGFLSGVF
>GhADK19
MIINKLSHLATVTAAAVTAGPPLYRLLKLTVSRFHGAAQPQPDTDYWYYYHVSPSQQESRRDELFRST
PVADTNGSVPLRGVQWAFIGSPRAKKRVYAVMLSKLLEVPHITMASLVRQELSPNSNLYKQIANAVD
HGEPVNEDIILGLLSKRLEDGHYRGETGFILDGIPRSRIQAEILDQLAEIDLVVNFKCTEDLMMNNQGEA
SWTERLQDYIKQSKVVEDYYKNEKKLLEFQIGNARMETWRRLLTALHLQHINAARLLQKPTCFQNQT
GWTGNWLVDWSRKRLKAG
>GhADK20
MASSSSVNLEDVPSESLMTELLRRMKCATKPEKRLILIGPPGSGKGTQSPMIKDEYCLCHLATGDMLR
AAVAAKTPLGVKAKEAMDKGELVSDELVVGIIDEAMKKPSCQKGFILDGFPRTVGQAQMLDEMLEK
QGVKIDKVLDFAIDDSILEERITGRWIHPASGRSYHTKFAPPKVPGVDDVTGEPLIQRKDDTAAVLKSRL
EAFHKQTEPVIDYYWKKGVVAKLHAEKSPKEVTEEVQKVLS
>GhADK21
MAIACLPKLRLATATAVSVSTFLKPLRHGLRSFGSAAALELPDEDVYYEYEQAHRDRRSAQPKIDVDGS
ATDRGVQWVLIGEPGVKRHAYAERLSKLLEVPHISMGTLVRQELNPHSSLYKQIANAVNEGKLVPEDV
IFALLSKRLEEGYYGGENGFILDGIPRTRIQAEILDQITDIDLVVNFKCTEEQMLKSSESEVLHIGSSKNVG
TSWKKNVHVYSEQAKSVEDYYSKQKKLLNFQVSAAPADAWQGLLAALHLQHINALTSSQKLTA
>GhADK22
MATNSMNLEDIPSESLMSELLRRMKCASKPDKRLILIGPPGSGKGTQSPIIKDEYCLCHLATGDMLRAA
VAAKTPLGIKAKEAMDKGELVSDDLVVGIIDEAMKKPSCKKGFILDGFPRTVGQAQKLDDMLGKQGV
KIDKVLDFAIDDAVLEERITGRWIHPASGRTYHTKFAPPKVPGLDDVTGEPLVQRKDDTAAVLKSRLDA
FHKQTQPVIDYYSEKGIVAKLHAEKPQKEVTDEVKKVLS
>GhADK23
MAVLSRCSRAVTAATTPNAAAAKFSYLIRALSPFSSSSSSTFDADNGLNSRIKPAVLGTLKREPKDRNV
QWVFLGCPGVGKGTYASRLSDLLGIPHIATGDLVRDELASSGPLSSQLKEIVNQGKLVSDEIIIDLLSKRL

EAGEAKGESGFILDGFPRTVRQAEILEGVTDIDLVINLKLREEALLAKCLGRRICSECGGNYNVACIDIKA
ENGKPGMYMAPLPPPPQCASKLITRADDTEEVVKQRLRIYQAMTLPVEDFYRSRGKLLEFDLPGGVRE
SWPKLLHALNLEDEEDKQSAAA
>GhADK24
MAVVSRCSRAATAATTANATTAPTFAYLIRSLSPFSSSSSTFYPQNDLNNRNKPAVLGTLKRESKGRN
VQWVFLGCPGVGKGTYASRLSNLLGVPHISTGDLVRDELASSGPLSSQLKEIVTQGKLVSDEIIIDLLSK
RLEAGEAKGESGFILDGFPRTVRQAEILEGVTDIDLVINLKLREEALLAKCLGRRICSECGGNYNVACIDI
KAENGRPGMYMAPLPPPPQCASKLITRPDDTEEVVKQRLRIYQAMTRPVEDFYRSRGKLLEFDLPGGI
PESWPKLLCALNLEDREDKQSAAA
>GhADK25
MASSSVNLEEIPSESLMNELLRRMKCAPKPEKRLILIGPPGSGKGTQSAIIKDEHCLCHLAAGDMLRAA
VSAKTPLGIKAKEAMDKGELVSDDLVVGIIDEAMKKPSCKKGFILDGFPRTVAQAQKLDEMLERQGVK
IDKVLDFAIDDAVLEERISGRWIHPASGRSYHTKFAPPRVPGVDDVMTGEPLIQRKDDSAAVLKSRLES
FHRQTQPVINYYSKKGIVATLHAEKPLTDVTDEVRKVLS
>GhADK26
MWRRVALLSSSISSSFLAQTASRLNTWESLTTGIAQQAKGVIAPKEKTPFITFVLGGPGSGKGTQCIKIV
ETFGFAHLSAGDLLRREIASKSADGAMILNTIKEGKIVPSQVTVKLIQKEMELSDNHKFLIDGFPRTEENR
VSFEQIIGVEPNVVLFFDCPEEEMVKRVLNRNEGRVDDNLETIKKRLTVFQALSLPVINYYSEKGKLYTIK
AIGTEDEIFEQVRPIFAAFEPHTIPLTLHFHCSSKGKKNQKQKRRQFEHNKNTAPALPFPRSSPTPLFINN
KPFPQTKLQAVDSIVKDLEASVKKGIIIDSEIFSSLLETCYQLKSIDHGIAIHRLVPQNLLRKNTGISSKLLRL
YATAGRMESAHQVFDQMSKRNEYAFPWNSLISGYAELGQYEDALALYFQMEEEGVEPDRFTFPRAL
KACAGIGSIHVGQAVHRDVVRKGFGNDVFVLNALIDMYAKCGDIVKARRVFDSIACKDNISWNSML
TGYIRHGLLAGALQVFRGMIQEGFEPDSVTISTILSSFCSLKTAAQIHGWVLRRGIEWNTSVVNAMIVV
YSNLGKLDGASWLFQRMPERDIVSWNSIISGHSKNPEALLYFEQMVRSCTSPDSITFVAILSACAHLGL
VKDGERLFWLMRKKYGIDPRMEHYACMINLYGRAGLIDEAFNMIVERMEFEAGPTVWGAMLYACSV
HGNIQIGEIAGQKLFELEPDNQHNFELLMKIYSNAGRVEDAERVRKLMLDRGL
>GhADK27
MGAVNVANKDINVSLAEKKPRVVFVLGGPGSGKGTQCANIVEHFHYTHLSAGDLLRAEIKSGSENGT
MIQNMIKEGKIVPSEVTIKLLEKAMLESGNDKFLIDGFPRNEENRAAFEAVTKIEPEFVLFFNCPEEEMEK
RLLSRNQGREDDNIETIRKRFKVFLDSSLPVIEYYKVKGKVREIEAAKPIGEVFEAVKVVFTPKAEKVKKSI
GLSLFTLETNRASSTHGRYALHIQVVA
>GhADK28
MADSAVALEDVASVDIMTELLRRFKCSSKPDKRLILIGPPGSGKGTQSPMIKDDYCLCHLATGDMLRA
AVAAKTPLGIKAKEAMDKGELVSDDLVVGIIDEAMKKTSCQKGFILDGFPRTVVQAQKLDEMLEKQG
TKIDKVLNFAIDDSILEERITGRWIHPSSGRTYHTKFAPPKVPGLDDVTGEPLIQRRDDTPAVLKSRLEAF
HRQTEPVIHYYGSKGILANLPAEKPPKEVTSEVQKVLSS
>GhADK29
MAVVSRCSRAATAATTANATTATTFAYLIRSLSPFSSSSSTFDPQNDLNNRNKPAVLGTLKRESKGRN
VQWVFLGCPGVGKGTYASRLSNLLGVPHIATGDLVRDELASSGPLSSQLKEIVTQGKLVSDEIIIDLLSK
RLEAGEAKGESGFILDGFPRTVRQAEILEGVTDIDLVINLKLREEALLAKCLGRRICSECGGNYNVACINI
KAENGRPGMYMAPLPPPPQCASKLITRPDDTEEVVKQRLRIYQAMTRPVEDFYRSRGKLLEFDLPGGI
PESWPKLLCALNLEDREDKQSAAA
>GhADK30
MTSSSVNLEEIPSESLMNELLRRMKCAPKPDKRLILIGPPGSGKGTQSPIIKYEHCLCSLATGDMLRAAV

SAKTPLGIKAKKAMDKGELISDDLVVGIIDEAMNKPSRKKGFILDGFPRTVAQAQKDHLHHKIAQCYIS
TGEKVLAIQFIRKTSTKGAGIFSGANRSVTEQMYEYGKNLGLLFQVVDDILDFTQSAEQLGKPAASASP
STSTRRKPKSAWNLIAWTQARASAANVGPPDTQFGPYCNGKRAVFKSMICGLTAINASRAVGCISKS
SLVVIQSAYPIARLFRAGCTIPLAPAESTQPWTASTLKTSRS

G. raimondii.

>GrADK1
MIINKLSHLATVTAAAVAAGPPLYRLLKLTVSRFHGAAQPQPDTDYWYYYHESPSQQESRRDELFRST
PVADANGSVPLRGVQWAFIGSPRAKKRVYAVMLSKLLEVPHITMASLVRQELSPNSNLYKQIANSVD
HGEPVNEDIILGLLSKRLEDGHYRGETGFILDGIPRSRIQAEILDQLAEIDLVVNFKCTEDLMMNNQGEA
SWTERLQDYIKQSKVVEEYYKNEKKLLEFQIGNARMETWRRLLTALHLQHINAARLLQKPTCFQNQTS
WTGNWLVDWSRTRLKAG

>GrADK2
MASSSSVNLEDVPSESLMTELLRRMKCATKPEKRLILIGPPGSGKGTQSPMIKDEYCLCHLATGDMLR
AAVAAKTPLGVKAKEAMDKGELVSDELVVGIIDEAMKKPSCQKGFILDGFPRTVGQAQMLDEMLEK
QGVKIDKVLDFAIDDSILEERITGRWIHPASGRSYHTKFAPPKVPGVDDVTGEPLIQRKDDTAAVLKSRL
EAFHKQTEPVIDYYWKKGVVAKLHAEKSPKEVTEEVQKVLS

>GrADK3
MAIARLPKLRLATATAVSVSTFLKPLRHGLRSFGSAAALELPDEDVYYEYEQAHRDRRSAQPKIDVDGS
ATDRGVQWVLIGEPGVKRHAYAERLSKLLEVPHISMGTLVRQELNPHSSLYKQIANAVNEGKLVPEDV
IFALLSKRLEEGYYGGENGFILDGIPRTRIQAEILDQITDIDLVVNFKRTEEQMLKSSESEVLHIGSSKDVG
TSWKKNVHVYSEQAKSVEDYYSKQKKLLNFQVSGAPADAWQGLLAALHLQHINALASSQKLTA

>GrADK4
MADSAVALEDVASVDIMTELLRRFKCSSKPDKRLILIGPPGSGKGTQSPMIKDDYCLCHLATGDMLRA
AVAAKTPLGIKAKEAMDKGELVSDDLVVGIIDEAMKKPSCQKGFILDGFPRTVVQAQKLDEMLGKQG
AKIDKVLNFAIDDSILEERITGRWIHPSSGRTYHTKFAPPKVPGLDDVTGEPLIQRKDDTPAVLKSRLEAF
HRQTEPVIDYYGSKGILANLPAEKPPKEVTSEVQKVLSS

>GrADK5
MATNSMNLEDIPSESLMSELLRRMKCASKPDKRLILIGPPGSGKGTQSPIIKDEYCLCHLATGDMLRAA
VAAKTPLGIKAKEAMDKGELVSDDLVVGIIDEAMKKPSCKKGFILDGFPRTVGQAQKLDDMLGKQGV
KIDKVLDFAIDDAVLEERITGRWIHPASGRTYHTKFAPPKVPGLDDVTGEPLVQRKDDTAAVLKSRLDA
FHKQTQPVIDYYSEKGIVAKLHAEKPQKEVTDEVKKVLS

>GrADK6
MASGSVNLEEIPYESLMNELLRRMKCAPKPEKRLILIGPPGSGKGTQSPIIKDEHCLCHLAAGDMLRAA
VSAKTPLGIKAKEAMDKGELVSDDLVVGIIDEAMKKPSCKKGFILDGFPRTVAQAQKLDEMLERQGVK
IDKVLDFAIDDAVLEERISGRWIHPASGRSYHTKFAPPRVPGVDDVTGEPLIQRKDDSAAVLKSRLESFH
RQTQPVINYYSKKGIVATLHAEKPLTDVTDEVRKVLS

>GrADK7
MWRRVASLSSSVSSSFLAQTASRLNTWESLTTGIAQQAKGVIAPKEKTPFITFVLGGPGSGKGTQCIKI
VETFGFAHLSAGDLLRREIASKSADGAMILNTIKEGKIVPSQVTVKLIQKEMELSYNHKFLIDGFPRTEEN
RVSFERIIGVEPNVVLFFDCPEEEMVKRVLNRNEGRVDDNLETIKKRLTVFQALSLPVINYYSEKGKLYTI
KAIGTEDEIFEQVRPIFAAFEKRPSL

>GrADK8
MLIMGSTSFSPSLSLSPSLSPHSSKFPNPNPNLFLRSHSSFTPLTLRSNQTRYRFKKLPSSNAPNFLVAAS
GKAEPLKVIISGAPASGKGTQCELITQKYGLVHIAAGDLLRAEVAAASENGKLAKEYMEKGELVPNEIV

VMMVKERLLQPDSQQRGWLLDGYPRSSSQAAALEDYGIRPDVFILLDVSEDILVERVVGRRLDPLTGK
IYHLKYSPPENDEIASRLTQRFDDTEEKVKLRLRTHHQNVDAVLSVYKDITVKVNGNEAKEHVFAQIDA
ALTHVGEQRKVNSGSLAA
>GrADK9
MLATSSSSSSSTAISLHPLSNYNASATPSLNPSSLSFSSSYSYLSPLSFRLHSSKTHLRITPKKGLRVSCST
NEPLKVMISGAPASGKGTQCELIVQKFGLVHISTGDLLRAEVSSGTEIGNKAKEFMNSGRLVPDEIVTA
MVTVRLSHQDAKEKGWLLDGYPRSFAQAQSLEELNIRPDIYIVLDVPDEILIDRCVGRRLDPVTGKIYH
LKNFPPESEEIKARLVTRADDTEEKVKSRLEIYKQNAAAISSTYSSITNKIDGNRPKEMIFKDIDSLLSQLLK
DKTVKSMKPVLQTESRLDQASSNQEKWRGIPTRLNNIPHSREIRNYFYDDVLQATQRAVNDGRTRLK
VEINIPELNPEMDVYRIGTLMELVRVIALSFADDGKRVKVCVQGSMGEGALAGMPLQLAGTRKILEFM
DWGNYGAMGTFIKIGSIGAKEVDEEDDMFILVAPQNAVGNCIVDDLRAMTDAAGKRPVILINPRLKD
LPGSSGIMQTMGRDKRLEYAASFESCYFFRLLYYAGTQYPIMGAIRMTYPYDYELYKRVDEPSGKEKYV
SLSTFKERPTIDEINDAFLGKPRNKDKKASGIWGFLSGVF
>GrADK10
MAVLSRCSRAVTAATTPNAAAAKFSYLIRALSPFSSSSSSTFDADNGLNSRIKPAVLGTLKREPKDRNV
QWVFLGCPGVGKGTYASRLSDLLGIPHIATGDLVRDELTSSGPLSSQLKEIVNQGKLVSDEIIIDLLSKRL
EAGEAKGESGFILDGFPRTVRQAEILEGVTDIDLVINLKLREEALLAKCLGRRICSECGGNYNVACIDIKA
ENGKPGMYMAPLPPPPQCACKLITRADDTEEVVKQRLRIYQAMTLPVEDFYRSRGKLLEFDLPGGVRE
SWPKLLHALNLEDEEDKQSAAA
>GrADK11
MAVVSRCSRAATAATTANATTATTFAYLIRSLSPFSSSSSSSTFDPQNDLNNRNKPAVLGTLKRESKGR
NVQWVFLGCPGVGKGTYASRLSNLLGVPHISTGDLVRDELASSGPLSSQLKEIVTQGKLVSDEIIIDLLS
KRLEAGEAKGESGFILDGFPRTVRQAEILEGVTDIDLVINLKLREEALLAKCLGRRICSECGGNYNVACID
IKAENGRPGMYMAPLPPPPQCASKLITRPDDTEEVVKQRLRIYQAMTRPVEDFYRSRGKLLEFDLPGGI
PESWPKLLCALNLEDREDKQSAAA
>GrADK12
MGTANDSSKDISVRLAEKNPKVVFVLGGPGSGKGTQCEKIVQHFGYTHLSAGDLLRAEKNSGSENGI
MIQNMMNEGKIVPSEVTIKLLQKAMLESGNDKFLIDGFPRNEENRAAFEAVTKIEPEFVLYFNCSEKEM
ERRILNRNQGREDDNIETIRKRFKVFLESSLPVIEYYKGKGKVREIDAAKPVEEVFEEVKDIFTPEVRKVVG
GRGWGRGCCAIL
>GrADK13
MAWLPKLRVATASAASSAVNPLRRWIRDIGSAAALELDYDYDCYDYERAPLDRRLPQQKLDVDGSV
PERGVQWVLIGEPGVKRHSYAERLSKLLGIPHISMGSLIRQELDPYSSLYKQVVNAVNEGKLVPEDVVF
ALLSKRLEEGENGFILDGIPRTRLQAEILDQIVDIDLVVNFKCSEEYMLKMSSESELLHIGNSKGVESSLKK
KLRVYSEQAKSVEDYYSKQKKLLNFQLAGATGDAWQGLLAALHLQYSPINAFSSSPKLTA
>GrADK14
MGAVNVANKDINVSLAEKKPRVVFVLGGPGSGKGTQCANIVEHFHYTHLSAGDLLRAEIKSGSENGT
MIQNMIKEGKIVPSEVTIKLLEKAMLESGNDKFLIDGFPRNEENRAAFEAVTKIEPEFVLFFNCPEEEMEK
RLLSRNQGREDDNIETIRKRFKVFLDSSLPVIEYYKAKGKVREIEAAKPIGEVFEAVKVVFTPKAEKVVA
Arabidopsis (*Arabidopsis thaliana*)
>AT5g50370
MATSSAASVDMEDIQTVDLMSELLRRMKCASKPDKRLVFIGPPGSGKGTQSPVIKDEFCL
CHLSTGDMLRAAVAAKTPLGVKAKEAMDKGELVSDDLVVGIMDEAMNRPKCQKGFILD
GFPRTVTQAEKLDEMLNRRGAQIDKVLNFAIDDSVLEERITGRWIHPSSGRSYHTKFAPPK

VPGVDDLTGEPLIQRKDDNADVLRSRLDAFHKQTQPVIDYYAKKENLVNIPAEKAPEEVT
KVVKKVVST
>AT5g63400
MATGGAAADLEDVQTVDLMSELLRRLKCSQKPDKRLIFIGPPGSGKGTQSPVVKDEYCLC
HLSTGDMLRAAVASKTPLGVKAKEAMEKGELVSDDLVVGIIDEAMNKPKCQKGFILDGFP
RTVTQAEKLDEMLKRRGTEIDKVLNFAIDDAILEERITGRWIHPSSGRSYHTKFAPPKTPGV
DDITGEPLIQRKDDNADVLKSRLAAFHSQTQPVIDYYAKKAVLTNIQAEKAPQEVTSEVK
KALS
>AT5g47840
MTGCVNSISPPPVTLYRHRASPSRSSFSLSGDALHSLYRHRRVSRSPSIIAPKFQIVAAEKSEP
LKIMISGAPASGKGTQCELITHKYGLVHISAGDLLRAEIASGSENGRRAKEHMEKGQLVPD
EIVVMMVKDRLSQTDSEQKGWLLDGYPRSASQATALKGFGFQPDLFIVLEVPEEILIERVV
GRRLDPVTGKIYHLKYSPPETEEIAVRLTQRFDDTEEKAKLRLKTHNQNVSDVLSMYDDIT
IKIEGNRSKEEVFAQIDSSLSELLQERNTAPSSLLS
>AT5g35170
MASLSLSSAHFSSTSSSSRSSISTSSLSPSSTSLPLLQSPIRRRYRSLRRRLSFSVIPRRTSRSFS
TSNSQIRCSINEPLKVMISGAPASGKGTQCELIVHKFGLVHISTGDLLRAEVSSGTDIGKRA
KEFMNSGSLVPDEIVIAMVAGRLSREDAKEHGWLLDGFPRSFAQAQSLDKLNVKPDIFILL
DVPDEILIDRCVGRRLDPVTGKIYHIKNYPPESDEIKARLVTRPDDTEEKVKARLQIYKQNS
EAIISAYSDVMVKIDANRPKEVVFEETQTLLSQIQLKRMIKTDKASPVQDKWRGIPTRLNN
IPHSRDIRAYFYEDVLQATIRSIKDGNTRLRVDINIPELNPEMDVYRIGTLMELVQALALSFA
DDGKRVKVCVQGSMGEGALAGMPLQLAGTRKILEYMDWGDDETLGTFVKLGAIGGKE
VDEEDDMFILVAPQNAVGNCIIDDLQAMTTAAGKRPVVLINPRLKDLPASSGIMQTMGRE
QRLEYALTFDNCYVFRLLYYLGTQYPIMGALRMSYPYRYELYKRVNEENGKEKYVLLATY
AERPTPEQIDDAFSGKSRDQSKKASGIWGFLSSVFS
>AT3g01820
MAWLSRVRGVSPVTRLAAIRRSFGSAAALEFDYDSDDEYLYGDDRRLAEPRLGLDGSGP
DRGVQWVLMGAPGAWRHVFAERLSKLLEVPHISMGSLVRQELNPRSSLYKEIASAVNERK
LVPKSVVFALLSKRLEEGYARGETGFILHGIPRTRFQAETLDQIAQIDLVVNLKCSEDHLVN
RNETALPQQEFLGSMLHSPVAINARRESVGVYAQEVEEYYRKQRKLLDFHVGGATSADT
WQGLLAALHLKQVNLTTSQKLTL
>AT2g39270
MAVSHRLLRPATTTIKNTFSSLFIRSLSSSSSGSSLDPKIDLEEAAAQLGKSSSTSTSPYKGRN
FHWVFLGCPGVGKGTYASRLSSLLGVPHIATGDLVREELSSSGLLSSQLKELVNHGKLVPD
EFIISLLSKRLQAGKDKGESGYILDGFPRTVTQAEILEGVTNIDLVINLKLREEALLAKCLGR
RICSECGGNYNVACIDIKGDDDTPRMYMPPLLPPPNCESKLISRADDTEEVVKERLRIYNK
MTQPVEEFYKKRGKLLEFELPGGIPESWARLLRALHLEDDKQSAIA
>AT2g37250
MARLVRVARSSSLFGFGNRFYSTSAEASHASSPSPFLHGGGASRVAPKDRNVQWVFLGCP
GVGKGTYASRLSTLLGVPHIATGDLVREELASSGPLSQKLSEIVNQGKLVSDEIIVDLLSKR
LEAGEARGESGFILDGFPRTMRQAEILGDVTDIDLVVNLKLPEEVLVDKCLGRRTCSQCGK
GFNVAHINLKGENGRPGISMDPLLPPHQCMSKLVTRADDTEEVVKARLRIYNETSQPLEE
YYRTKGKLMEFDLPGGIPESWPRLLEALRLDDYEEKQSVAA

Tomato (*Solanum lycopersicum*)

>SlADK1
MGTVVESANQGAVSLPTNKKVTVIFVLGGPGSGKGTQCANIVEHFGYTHLSAGDLLRAEI
KSGSENGTMISNMIKEGKIVPSEVTVKLLQRAIQENGNDKFLIDGFPRNEENRAAFELVTGI
EPEFVLFFDCPEAEMEKRLLGRNQGREDDNIETIKKRFNVYMESSLPVIEHYNSKGKVRKI
DAVKPVGEVFEAVKAVFAPSNEKVAA
>SlADK2
MAMLSFLGVSARTFLRAASSKSVRAYGSAVAAHFDYDNEEDMEEPSGSVPRRGVQWLIM
GHPMTQRHVYAQWLSKLMDVPYISMGSLVPQQLNPHYNKISSVVNEGKHVPEEVIFGLLS
KRLEEGHCRGENGFILDGIPRTMLQAEILDKVVDIDLVLNLKCSVSKNDRSNGIYSTEDQL
LKRGNLMSSRVMDGGAWKEKQYDHDEQIKPLEEYYRKQKKLLNYQVAGGPAETWQGL
LAALQLQHMMSAVGSTQLTAGC
>SlADK3
MALLSRIRAAAKPLIRTESLRSYGSAAAQLVDYDYDDYEYEEFQNRSCVMEESEGSVPRR
GVQWVIMGDPMAQRHVYAQWLSKLLGVPHISMGSLVRQELHPRSSLYKQIADAVNQGKL
VPEEVIFGLLSKRLEEGYCSGESGFILDGIPRSKIQAEILDKTVDIDLVLNLKRAEDLVSKKD
KSTGLYPPLEFLRMGASGISTSRQPEGGHFRPSSIMEDVSRKNLHVHAEQVNPLEEYYRKQ
RKLLDFQVAGGPGETWQGLLAALHLQHRNAVGSTQLTAGC
>SlADK4
MWRRRFTSLPLFFSHLQQVRRADELKICQAFCTETVKPPVEGESNSGRNSPFVAFVLGGPG
SGKGTQCLKIAETFGFDHIGAGDLLRKEMHSDSENGAMIQKLMKEGSIAPSEVTVKLIKK
AIESAENRKFLIDGFPRSEENRVAYERIIGAEPNFVLFFDCPEEVMVKRVLNRNEGRVDDNE
HTVKERLKVYKAITLPVANHYAMKGKLYKVDGTGTQEEIFERVRPIFASLRLST
>SlADK5
MSTSSVNLEDVPSESLMSELLRRMRCSSKPDKRLILIGPPGSGKGTQSPIIKDEYCLCHLAT
GDMLRAAVAAKTPLGIKAKEAMDKGELVSDDLVVGIIDEALKKPSCQKGFILDGFPRTVV
QAEKLDVMLQNRGTKVDKVLNFAIDDAILEERITGRWIHPASGRSYHTKFAPPKVPGIDDV
TGEPLIQRKDDTAAVLKSRLEAFHRQTEPVIDYYAKKGNVVNLPAEKPPQAVTAEVKKVL
S
>SlADK6
MVVWTRAVVRTWRCRPTNFSRAFSEKLPTSEPKGRNIQWVFLGCPGVGKGTYAARLSKL
LGVPHIATGDLVRQQLSSHGPLASKLVDIVSQGQLISDEIVIDLLSKRLEAGEAKGETGFILD
GFPRTIRQAEILEGVTDIDLVINLKLREDALIAKCLGRRTCSECGGNYNVACIDMKGDDGE
TRMYMPPLLPPPHCETKLITRSDDTENVVKERLRIYHEMSKPVEDFYRQRGKLLEFDLPG
GIPESWSKLLQALNIYDDEDKKSAAA
>SlADK7
MAASLEDVPSESLMSEVLRRLRCSSKPDKRLILIGPPGSGKGTQSPIIKDEYCLCHLATGDM
LRAAVAAKTPLGIKAKEAMNNGELVSDDLVVGIIDEAMKKPSCQKGFILDGFPRTVVQAE
KLDEMLQKQGSKIDKVLNFAIDDAILEERITGRWIHPSSGRSYHTKFQPPKVPGVDDVTGE
PLIQRKDDTAEVLKSRLDAFHRQTEPVINYYSTKGVVASLHAEKPPKEVTSEVKHVLSS
>SlADK8
MASCCSLSFSTVSSKPNKPYSSPISSSLELPFTSQLPFSKKYSLYSNHTLLQTQCRKTQSPDC
PSFLVVGSAKKQEPLRVMISGAPASGKGTQCELITKKYDLVHIAAGDLLRAEIAAGTENGR
RAKEYMDKGQLVPNEIVVTMVKERLMCPDSQEKGWLLDGYPRSLSQAVALKEFQPNLFI

LLEVPEEILVERVVGRRLDPVTGRIYHLKYSPPETDEIAARLTQRFDDTEEKVKLRLHTHR
QNVESVLSMYKDTIFQVDGSVSKEEVFAQIDGALTQLLEAKE
>SlADK9
MDLHKEGDTGSAKQKKVKIVFVIGGPGSGKGTQCKRIAQQFGYTHLSVGEILRQETSSGS
ETGHMVQKIMKEGKLVPSDVTVRLLQQAMQGIDNDKFLIDGFPRDEENVKAFEDLTKME
PEFVLYLDCPQDEMEKRLLSRNEGREDDNIETIRKRLKVFVESTLPTIEYYESKGKIRKVD
AGKSIDEVFESIKVIFSPGKDNKMPPSKHKCKCLIL
>SlADK10
MAAMIRLFRSSSSSSSLISRSLSTAAASETVKSRSYPHSTSVEPKAKSVQWVFLGCPGVGK
GTYASRLSTLLGVPHIATGDLVRDELKSSGPLSKQLAEIVNQGKLVSDEIILNLLSKRLESGE
AKGEAGFILDGFPRTVRQAEILTEVTDIDLVVNLKLPERVLVEKCLGRRICSECGKNFNVAS
IDVAGENGAPRISMAPLNPPSQCISKLITRADDTEAIVKERLSIYWDKSQPVEDFYRSQGKL
LEFDLPGGIPESWPKLLEVLNLDEQEHKLSAAA
>SlADK11
MAMIASVTMNFPHISTHNISSNQTFSPICTNNPSNFSSSSSSSIPISSNSIRLSSSIAYSEQLIAS
HNVNRRTKNRKIKVISARSEPLKVMISGAPASEKDVVGWMKSLYPLQEVSGLSNGSGEIS
GSERFLLQYWSYATQILFSQTNGLIEQQKFVFCGFGLVHISTGDLLRAELSAGTDIGNKAKE
YMNAGRLVPDEIVTAMVTTRLSKEDAKEKGWLLDGYPRTLAQAESLERLNIRPDIYIVLD
VPDAILIDRCVGRRLDPLTGKIYHVTNFPPETEDIKARLITRPDDTEEKVKSRLQIYKQNAE
AILPVYSDIMNKIDGNRGKDSVFAEIDSLLSRVQKEEQDARKSEESAISSTRADMASLSKD
WRGIPTRLNNIPHSREIREYFYTDVLQATQRAVNDGKTRLKIEINIPELNPSMDVYRIGTLM
ELIRVLALSFADDGKRVKVCVQGSMGEGALAGMPLQLAGSRKILEYMDWGDYGALGNFI
NIGSIGGKEVEKQDDVFILVAPQNAVGNCIIDDMRAMTDAAGNRPIILVNPKLKDLPASSGI
MQTMGRDKRLEYAALFEICYQFRLLYYAGTQYPIMGALRMSYPYPYELYKRVDESPGKE
KYISLATFAKRPSIDEMNDAFEGKSRNQEKKAEGFWYVINYPPFYLCF
Potato (*Solanum tuberosum*)
>Sotub04g013920
MVVWTRAVVRTWRCRPTNFSRAFSEKLPTPEAKGRNVQWVFLGCPGVGKGTYAARLSK
LLGVPHIATGDLVRQQLSSHGPLALKLVDIVSQGQLISDEIVIDLLSKRLEAGEAKGEIGFIL
DGFPRTIRQAEILEGVTDIDLVINLKLREDALIAKCLGRRTCSECGGNYNVACIDMKGDDG
ETRMYMPPLLPPPHCETKLITRSDDTENVVKERLRIYHETSKPVEDFYRKRGKLLEFDLPG
GIPESWQKLLQALNIYDDDEDKKSAAA
>Sotub09g006620
MAAMIRLFRSSSSSSSNSISLISRSLSTAAASETVKSQSYPHNPHSTSVDPKAKTVQWVFLG
CPGVGKGTYASRLSTLLGVPHIATGDLVRDELKSSGPLSKQLAEIVNQGKLVSDEIILNLLS
KRLESGEAKGEAGFILDGFPRTVRQAEILTEVTDIDLVVNLKLPERVLIEKCLGRRICSECG
KNFNVASIDVAGENGAPRISMAPLNPPSQCVSKLITRADDTEAIVKERLSIYWDKSQPVED
FYRSQGKLLEFDLPGGIPESWPKLLEVLNLDEQEYKLSAAA
>Sotub03g005270
MALLSRIRAAVQPLIRTESLRSYGSAAAQLVDYDYDDYEYEEYQNRSYVMEESEGSIPRR
GVQWVIMGDPMAQRHVYAQWLSKLLDVPHISMGSLVRQELHPRSSLYKQIADAVNQGKL
VPEEVIFGLLSKRLEEGYCSGESGFILDGIPRSKIQAEILDKTVDIDLVLNLKCAEDLVSKKD
KSTGLYPPLEFLRRGASGISTSRQPEGGHFRPSSIMDDVSRKNLHVHAEQVNPLEEYYRKQ
RKLLDFQVAGGPGETWQGLLAALHLQHRNAVGSTQLTAGC

>Sotub02g037180
MAMLSFLGVSARPFLLAASSKSVRAYGSAAAAHFDYDYEEEPSGSVPRRGVQWLIMGDP
MTQRHVYAQWLSKLVDVPYISMGSLVRQELNPHYNKISSVVNEGKLVPEEVIFDLLSKRL
EEGYCRGENGFILDGIPRTMFQAIKRLEEYYRKQKKLLNYQVAGGPAETWRGLLAALQLQ
HMMSAVGSTQLTAGC
>Sotub03g023880
MSTSSVNLEDVPSESLMSELLRRMRCSSKPDKRLILIGPPGSGKGTQSPIIKDEYCLCHLAT
GDMLRAAVAAKTPLGIKAKEAMDKGELVSDDLVVGIIDEALKKPSCQKGFILDGFPRTVV
QAEKLDVMLQSRGTKVDKVLNFAIDDAILEERITGRWIHPASGRSYHTKFAPPKAPGIDDV
TGEPLIQRKDDTAAVLKSRLEAFHRQTEPVIDYYAKKGNVVNLPAEKPPQAVTAEVKKVL
S
>Sotub05g016010
MAASLEDVPSESLMSEVLRRLRCSSKPDKRLILIGPPGSGKGTQSPIIKDEYCLCHLATGDM
LRAAVAAKTPLGIKAKESMNNGELVSDDLVVGIIDEAMKKTSCQKGFILDGFPRTVVQAE
KLDEMLQKQGAKIDKVLNFAIDDAILEERITGRWIHPSSGRSYHTKFQPPKVPGVDDVTGE
PLIQRKDDTAEVLKSRLDAFHRQTEPVINYYSTKGVVASLHAEKPPKEVTSEVKNVLSS
>Sotub01g028550
MGTVVESANQGAVSLPTNKKVTVIFVLDLFPLLTWTGGPGSGKGTQCTNIVEHFGYTHLS
AGDLLRAEIKSGSENGTMISNMIKEGKIVPSEVTIKLLQRAIQENGNDKFLIDGFPRNEENR
AAFELVTGIEPEFVLFFDCPEAEMEKRLLGRNQGREDDNIETIRKRFNVYMESSLPVIEYY
NSKGKVRKIDAVKPVGEVFEAVKAVFTPANEKVKY
>Sotub08g022760
MDLHKEGDRGSAKQKKVKIVFVIGGPGSGKGTQCKRIAQQFGYTHLSVGEILRQEISSGS
ETGSMIQKIMKEGKLVPSDVTVRLLQQAMQGINSDKFLIDGFPRNEENVKAFEDLTKMEP
EFVLYLDCPQDEMEKRLLSRNEGREDDNIETIRKRFKVFMESTLPTIEYYESKGKIRKVDA
GKSVDEVFESIKVIFSQGKDNKVPPSRHKCKCLIL
>Sotub11g015570
MASCSSLNFTAVSSNPQKLPSSSISSPIVQRPFTSHLSFSKSSSLHSDQIPIRTHCGKLPQPNG
AGFVVLGCARKKEPLRIMISGAPASGKGTQCELITQKYGLVHIAAGDLLRAEIAAGSENGK
QAKEYMDKGKLVPNEIVVTMVKERLNGPDSREKGWLLDGYPRSSSQAIALEEFGFQPDL
FILLEVPEEILVERVVGRRLDPITGKIYHLKYSPPETEEIASRVTQRFDDTEEKACIVKLRLQ
THHQNVEAILLMYEDITVKVNGIGSKQEVFAQIDGALTQLLEQKQEKLGTVAA
>Sotub06g024300
MASCCSLSFSTVSSKPNKPYSSPISSSLQLPFTSQLPFSKKKSLYSNHTLLQTQCRKTPSPDC
PSFLVVGSAKKQEPLRVMISGAPASGKGTQCELITKKYDLVHIAAGDLLRAEIAAGTENGR
RAKEYMDKGQLVPNEIVVTMVKERLMRPDSQEKGWLLDGYPRSSSQAVALKEFQPDLFI
LLEVPEEILVERVVGRRLDPVTGRIYHLKYSLPETDEIAARLTQRFDDTEEKVKLRLHTHH
QNVESVLSMYKDTIFQVDGSVSKEEVFAQIDAALTQLLEAKE
>Sotub03g020180
MWRRFTSLPLFFSHLQQVRRADELKICQAFCTEIVKPPVEGESNSRRNIPFVAFVLGGPGSG
KGTQCLKIAETFGFDHIGAGDLLRKEIHSDSENGAMIQKLMKEGSIAPSEVTVKLIKKAIES
AENRKFLIDGFPRSEENRVAYERIIGAEPNFVLFFDCPEEVMVKRVLNRNEGRVDDNEHTV
KERLKVYKAITLPVANHYAKKGKLYKVDGTGTQEEIFERVRPIFASLRLST
>Sotub12g007490

MDVYRIGTLMELIRVLALSFADDGKRVKVCVQGSMGEGALAGMPLQLAGSRKILEYMD
WGDYGALGNFVNIGTIGGKEVEKQDDLFILVAPQNAVGNCIIDDMRAMTDAAGNRPIILV
NPKLKDLPASSGIMQTMGRDKRLEYAASFEICYQFRLLYYAGTQYPIMGALRMSYPYPYE
LYKRVDESPGKEKYISLATFAKRPSIDEMNDAFDGKSRNQEKKAEGFWGFLSGIL

Rice (*Oryza sativa*)

>Os12t0236400
MAANLEDVPSMELMTELLRRMKCSSKPDKRVILVGPPGCGKGTQSPLIKDEFCLCHLATG
DMLRAAVAAKTPLGIKAKEAMDKGELVSDDLVVGIIDEAMKKTSCQKGFILDGFPRTVVQ
AQKLDEMLAKQGTKIDKVLNFAIDDAILEERITGRWIHPSSGRSYHTKFAPPKTPGLDDVT
GEPLIQRKDDTAAVLKSRLEAFHVQTKPVIDYYTKKGIVANLHAEKPPKEVTVEVQKALS
>Os11t0312220
MAAAANLEDVPSMDLMNELLRRMKCSSKPDKRLILVGPPGSGKGTQSPIIKDEYCLCHLA
TGDMLRAAVAAKTPLGVKAKEAMDKGELVSDDLVVGIIDEAMKKPSCQKGFILDGFPRT
VVQAQKLDEMLEKKGTKVDKVLNFAIDDSILEERITGRWIHPSSGRSYHTKFAPPKVPGV
DDVTGEPLIQRKDDTAEVLKSRLEAFHKQTEPVIDYYSKKALVANLHAEKPPKEVTAEVQ
KVLS
>Os08t0288200
VHISTGDLLRAEVSSGTEIGKKAKEYMDNGMLVPDQVVTDMVVSRLSQPDVRERGWLL
DGYPRSYAQAQSLESMKIRPDIFIVLEVPDDILIDRCVGRRLDPETGKIYHIKNFPPENDEVS
ARLVTRSDDTFEKVKSRLDTYKQNSEAVIPTYSDLLNQIDGNRQVEVVFNEIDSLLQKICE
NASFNMLAKTNGKPQDSKDTTASKNEFRGIPTRLNNIPHSREIRKYFYNDVLVATRHAVED
KKTRLQIDINIPELNPEMDVYRIGTLMELVRELSLSFADDGKRVKVCVQGSMGQGAFAGIP
LQLAGTRKILEIMDWGEYGAKGTFINFGAVGASEVDKEDDMFILIAPQNAVGNCIIDDMK
AMTDAAGDRPVILVNPRLKDMPGSSGVMQTMGRDMRLKYAASFETCYSFRLLFYAGSFY
PIMGALRMAYPNKYEIYRRVDEPNGQERYVLLEEFVEKPTPDEITNAFRPRKNENEKSASG
FWGFLSGIL
>Os08t0118900
MAGVLRLAGAARSPLARALAPAARRMGASAAAAMEDEAYWTEWEEEEEKARARESAP
VAEMCPTGGGGGGPQWVVMGRPGPQKHAHAARLAEVLAVPYISMGTLVRQELSPASSLY
KKIANSVNEGKLVPEDIIFGLLTKRLEEGYNKGETGFILDGIPRTHMQAEILDEIVDIDLVLN
FKCADNCFMKRRFGGDICPHCGQLFDFSKTASSDRNPSLGSCTWPSQVQHAAVLGLEDSR
MEKMRAYAEQTKLLEDYYRKQRKLMELKTSARPGETWQGLVAALHLQHLDASPTPHKL
TM
>Os08t0109300
MASSMAATATLSPPVLSAERPTVRGGLFLPPSPATSRSLRLQSARRCGISPATRKPRSLPRAA
KVVVAVKADPLKVMIAGAPASGKGTQCELIKSKYGLVHISAGDLLRAEIAAGSENGKRAK
EFMEKGQLVPDEIVVNMVKERLLQPDAQEKGWLLDGYPRSYSQAMALETLNIRPDIFILL
DVPDELLVERVVGRRLDPVTGKIYHLKYSPPENEEIASRLTQRFDDTEEKVKLRLQTHYQN
VESLLSIYEDVIVEVKGDALVDDVFAEIDKQLTSSLDKKTEMVASA
>Os07t0412400
MASRGGGARRTRPNVLVTGTPGTGKTTTCSLLADAVDLRHINIGDLVREKSLHDGWDEEL
ECHIINEDLVCDELEDVMEEGGILVDYHGCDFFPERWFDLVVVLQTDNSILHDRLTSRGYM
GAKLTNNIECEIFQMLLEEARESYKEEIVMPLRSDNVEDISRNVGTLTEWINNWRPSRS
>Os03t0130400

MAAVQRLLRASASGGAAAAAAAARRRMSTAVAPEQTPAAAAFPFAAAAGRARQRVAEER
NVQWVFLGCPGVGKGTYASRLSRLLGVPHIATGDLVRDELASSGPLSVQLAEIVNQGKLV
SDEIIINLLSKRLKKGEEQGESGFILDGFPRTVKQAEILDGVTDIDMVVNLKLREDVLVEKC
LGRRICGQCGKNFNLACIDVKGENGLPPIYMAPLLPPNNCMSKLITRADDTEEVVRNRLQI
YNDMSQPVEGFYRQQGKLLEFDLPGGIPESWPKLLHVLNLEDQEEMKLATA
