# Supplementary figures and images for: Genome-Wide Identification and Expression Analysis of ADK Gene Family Members in Cotton under Abiotic Stress
Source: Int J Mol Sci. 2024 Jul 17;25(14):7821. doi: 10.3390/ijms25147821 (PMC11277214; doi:10.3390/ijms25147821)

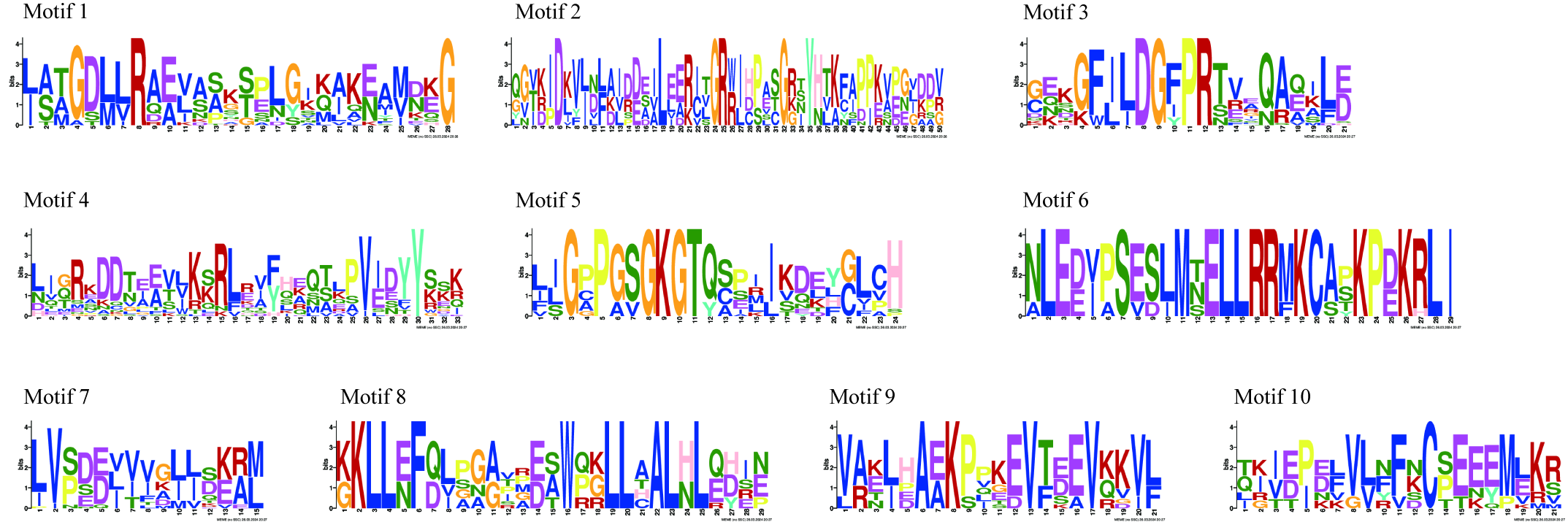

Supplement: Supplementary file 1 [file ijms-25-07821-s001.zip › Additional file S2.tif]
